# Microbial Diversity of the Baikal Rift Zone Freshwater Alkaline Hot Springs and the Ecology of Polyextremophilic Dissimilatory Iron-Reducing Bacteria

**DOI:** 10.3390/biology14121716

**Published:** 2025-12-01

**Authors:** Anastasia I. Maltseva, Alexander G. Elcheninov, Alexandra A. Klyukina, Alexandra V. Gololobova, Elena V. Lavrentyeva, Tuyana G. Banzaraktsaeva, Vyacheslav B. Dambaev, Darima D. Barkhutova, Daria G. Zavarzina, Evgenii N. Frolov

**Affiliations:** 1 Winogradsky Institute of Microbiology, Federal Research Center of Biotechnology, Russian Academy of Sciences, Moscow 117312, Russia; elcheninov.ag@gmail.com (A.G.E.); alexandra.a.popova@gmail.com (A.A.K.); sasha.gololobova@yandex.ru (A.V.G.); zavarzinatwo@mail.ru (D.G.Z.); evgenii_frolov_89@mail.ru (E.N.F.); 2 Institute of General and Experimental Biology, Siberian Branch of the Russian Academy of Sciences, Ulan-Ude 670047, Russia; lena_l@mail.ru (E.V.L.); tuyana_banz@mail.ru (T.G.B.); astra78plus@mail.ru (V.B.D.); darima_bar@mail.ru (D.D.B.)

**Keywords:** Baikal rift zone, hot springs, iron-reducing microorganisms, microbial communities, 16S rRNA gene metabarcoding, thermophile

## Abstract

Polyextremophilic microbial communities of Baikal Rift Zone hot springs have been studied fragmentarily, and these studies have typically focused on either phototrophic microbial mats or on the whole microbial community from one or a few sites. In our work, we conducted the first large-scale screening of microbial communities from seven hot spring groups in the Baikal Rift Zone, using metabarcoding of the V3-V4 regions of the 16S rRNA gene. Analysis of alpha and beta diversity, as well as co-occurrence network analysis, revealed that the microbial diversity of the studied springs is highly dependent on temperature values. This approach allowed classifying microbial communities into four distinct groups, characterized by significantly different taxa representing the key functional roles of primary producers, heterotrophic consumers, and terminal destructors of organic matter. Sulfate-reducing bacteria constituted a major metabolic group driving the final stage of organic matter mineralization. Moreover, the presence of alkalithermophilic dissimilatory iron reducers, whose existence was debatable, was proved in the studied samples by cultural methods. The phylotypes that gained an advantage on selective media with synthesized ferrihydrite and hydrogen or acetate added as an electron donor belonged to the genus *Parvivirga* of the order *Anaerosomatales* and several unknown representatives of the phylum *Bacillota*.

## Simple Summary

This article presents the results of the first large-scale survey of microbial diversity in freshwater alkaline hot springs in the Baikal Rift Zone, revealing through 16S rRNA gene metabarcoding and statistical methods that temperature is the primary factor influencing the microbial community structure. The phyla were distributed between the two groups. The first small group included *Armatimonadota*, *Deinococcota*, *Aquificota* and DRYD01, which were integral components of the microbial communities of the hot springs with the highest temperatures (58–74 °C), i.e., these were extremely thermophilic and thermophilic microbial communities with low biodiversity. The second-largest group comprised the phyla *Pseudomonadota*, *Nitrospirota*, *Desulfobacterota*, *Actinomycetota*, *Verrucomicrobiota*, *Spirochaetota*, *Bacillota*, *Bipolaricaulota* (*Acetothermia*), *Bdellovibrionota*, *Thermoproteota*, *Hadarchaeota*, and others, which were part of more diverse, moderately thermophilic and mixed microbial communities of thermal springs with lower temperatures from 32 to 59 °C. Classical microbiological methods were used to culture and identify a physiological group of prokaryotes previously debated to exist—alkalithermophilic dissimilatory iron-reducing microorganisms. The obtained enrichment cultures were dominated by novel lineages of *Bacillota* and *Actinomycetota*, including the genus *Parvivirga*. Our findings expand the knowledge about microbial life in polyextreme environments and the understanding of its role in biogeochemical cycling in the studied ecosystems of the Baikal Rift Zone.

## 1. Introduction

The Baikal Rift Zone (BRZ) is a large Cenozoic continental rift located in Asia that frames the Siberian Craton to the south and southeast. It extends for approximately 2500 km from northwest Mongolia, through the East Siberian Mountains, to the Alban Shield. The central segment of the BRZ, located immediately along the craton margin, is represented by the Lake Baikal Basin. This basin is both the largest and the oldest part of the system, making up approximately one-third of the entire rift zone and extending for about 680 km.

Due to the combination of complex geological features, such as modern tectonic activity and high seismic activity, as well as the presence of deep faults that crack Proterozoic granites, gneisses, and gneissoid granites, making them permeable to meteoric waters, the BRZ is characterized by a large number of springs with various physical, chemical, and gas properties [1]. Low mineralization (usually up to 1 g/L) and a neutral or alkaline pH are typical of springs in the BRZ region, as well as a wide range of temperatures [2] and nitrogen saturation [1,3]. Most researchers agree that hot springs of the BRZ region form through the infiltration of cold meteoric water along fractures, where it interacts with the surrounding rocks, leading to changes in its cationic, anionic, and gaseous compositions [4]. All hydrotherms of the BRZ are divided into five groups according to the chemical composition: sodium hydrocarbonate (HCO_3_–Na), sodium fluoride–hydrocarbonate (HCO_3_–F–Na), sodium sulfate–hydrocarbonate spring (HCO_3_–SO_4_–N), sodium hydrocarbonate–sulfate spring (SO_4_–HCO_3_–Na), and sodium sulfate (SO_4_–Na) types [5].

The Barguzin Valley (BV) is one of the largest depressions in BRZ, located in the Republic of Buryatia (Russia), and extends for about 200 km. All hot springs in this area are alkaline (Supplementary Materials S1 and Table S1 of the Supplementary Materials S2) [4], which contributes to the development of polyextremophilic alkalithermophilic microorganisms. Temperature and pH are not the only environmental factors that shape the microbial diversity of hot springs. Redox potential, trace element levels, organic matter composition, geochemistry of hydrothermal fluids, solar radiation budgets, and trophic relationships also play a crucial role [6].

Thermal fields in the Baikal region are located not only in BV, but also along the shore of Lake Baikal as a part of active balneological resorts in Goryachinsk and Zmeinaya Bay. Balneological resorts include thermal baths with alkaline water at a temperature of around 47 °C. In previous years, microbial communities of the BRZ hot springs have been actively studied using microbiological and molecular techniques [3,4,7,8,9,10]. However, the most comprehensive studies were made with samples of cyanobacterial communities and anoxygenic phototrophic bacteria, which are diverse and represent the primary producers in hot springs of the BRZ [2,4,8,9,10,11,12,13,14,15,16]. Research on chemotrophic anaerobic microorganisms, which are responsible for both the primary production of organic matter and its mineralization, has been conducted only on a fragmentary basis [17,18]. In particular, nothing was known about alkalithermophilic dissimilatory iron reducers, whose existence had not been proven but was thermodynamically justified by Nixon et al. [19]. Our study of the Goryachinsk thermal field (pH 9.0, 50–55 °C) conducted in 2024 revealed the presence of alkalithermophilic iron reducers in all the samples collected. Additionally, the first enrichment cultures with chemolithotrophic representatives of this group were obtained. Furthermore, two distinct ecological niches were identified for these microorganisms in the Goryachinsk thermal field: (i) a surface niche rich in organic matter, and (ii) a subsurface one poor in it [19].

Based on these findings, we conducted a more extensive and targeted search for this ecophysiological group in the sediments of Barguzin Valley’s hot springs. During the 2024 expedition, we collected samples of sediments and water from seven thermal fields and springs, including Goryachinsk and Zmeinaya Bay. We characterized the composition of microbial communities using molecular methods and performed correlation analysis to identify a link between the microbial community structure and key physicochemical parameters of water (pH, Eh, and temperature). To isolate pure cultures of dissimilatory alkalithermophilic iron reducers, we carried out enrichments using molecular hydrogen or sodium acetate as electron donors and synthesized ferrihydrite as the electron acceptor. The results are presented in this paper.

## 2. Materials and Methods

### 2.1. Sample Collection

**Figure 1:**
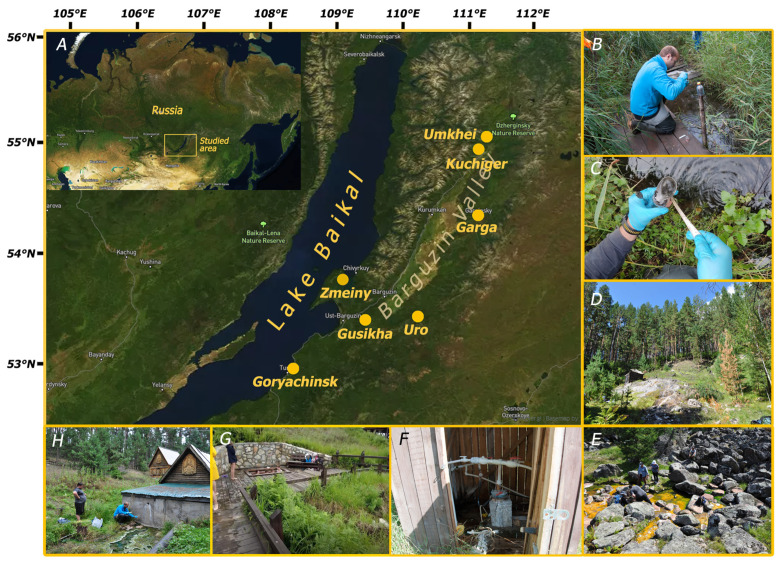
Location of the sampling sites in BRZ (**A**). Photographs of the thermal groups and springs: Umkhei (**B**), Kuchiger (**C**), Garga (**D**), Uro (**E**), Gusikha (**F**), Zmeiny (**G**), and Goryachinsk (**H**).

The samples of microbial mats, sediments beneath the mats, and biofilms mixed with thermal water were collected with clean spatulas into 50 mL glass vials (Figure 1C), which were filled with the sample with no headspace left and closed with rubber stoppers, crimped with aluminum caps and stored at +4 °C in fridge until transported to the laboratory (Supplementary Materials S1). During transportation, the samples were also maintained at +4 °C. The pH and temperature were measured in situ using SG2 SevenGo™ pH meter (Mettler-Toledo, Greifensee, Switzerland) with InLab^®^ Expert Go sensor (Mettler-Toledo, Greifensee, Switzerland), while the Eh values were measured using 301Pt-C ORP combination electrode (Apera Instruments, Shanghai, China).

### 2.2. Enrichment Cultures

To obtain enrichment cultures of alkalithermophilic iron reducers, we focused on profiling data reflecting the taxonomic diversity in the sample, the visual presence of organic sediment that interferes with the isolation of chemoautotrophic microorganisms, as well as the description of the sample, where iron reducers could potentially be, temperature and pH, which are best suited for the isolation of this group of microorganisms (Table 1 and Supplementary Materials S1). The selected samples were as follows: #4710 (Garga thermal field), #4722 (Uro thermal field), #4725, #4730 (Gusikha thermal field), and 4731-4733 (Goryachinsk thermal field). Enrichment cultures were initiated by inoculating anoxic sterile liquid medium with 10% (*v*/*v*) sample. The medium was prepared under 100% N_2_ gas atmosphere and had the following composition (g/L): KH_2_PO_4_, 0.2; MgCl_2_·2H_2_O, 0.1; NH_4_Cl, 0.5; KCl, 0.2 and Na_2_CO_3_, 0.5. Solutions of vitamins [20] and trace elements [21] were added (1 mL/L each) after sterilization. Sodium acetate (10 mM) or molecular hydrogen (10% *v*/*v*) was used as the substrate. Synthesized ferrihydrite prepared as described previously [22] was used as an electron acceptor at a final concentration of Fe(III) −25 mM. The pH of the medium was adjusted with 10% NaOH to the pH value of the thermal springs at the time of sampling (Table 1 and Supplementary Materials S1). No reducing agents were added to the medium. The medium (10 mL) was dispensed into 17 mL Hungate tubes under the flow of N_2_. Incubation of the enrichment cultures was carried out at the temperatures detected on the sample sites (Table 1 and Supplementary Materials S1). Growth of the enrichment cultures was monitored using a fluorescence microscope, Axio Lab.A1 (Zeiss, Oberkochen, Germany), from which subsamples were pre-stained with acridine orange dye for DNA.

### 2.3. DNA Extraction and 16S rRNA Gene Sequencing

For DNA isolation, environmental samples were taken aseptically in 2 mL Eppendorf tubes with screw caps and then fixed with a stabilizing buffer (100 mM EDTA, 100 mM Tris-HCl, 150 mM NaCl). During transportation and storage, the fixed samples were maintained at 4 °C and then stored at −20 °C until DNA was extracted.

For DNA extraction from the enrichments, we collected enrichment cultures that showed ferrihydrite reduction. For this purpose, the Hungate tubes were shaken, and the suspension (5 mL) was collected with a syringe and centrifuged for 20 min at 14000 g and 4 °C. The supernatant was discarded, and the pellet was used to extract total DNA.

DNA from the environmental samples and enrichment cultures was extracted using the FastDNA™ SPIN Kit for Soil (MP Biomedicals, Santa Ana, CA, USA) according to the manufacturer’s instructions.

Amplicon libraries of the V3-V4 hypervariable region of the 16S rRNA gene were prepared according to Gohl et al. [23] with the following primers: 341F (5′-CCTAYGGGDBGCWSCAG-3′) and 806R (5′-GAC TAC NVG GGT MTC TAA TCC-3′) [24,25], including Illumina technical sequences [26]. The libraries were prepared and sequenced in two replicates for each sample. High-throughput sequencing of the libraries was performed with MiSeq Reagent Kit v2 (500-cycles) (Illumina, San Diego, CA, USA) on a MiSeq sequencer (Illumina, San Diego, CA, USA) according to the manufacturer’s instructions.

### 2.4. Data Analysis

Primary raw reads were processed as described earlier [26]. Reads obtained after adapters and primers trimming were filtered (with the following parameters: truncLen = 380, maxN = 0, maxEE = 2, and truncQ = 2) and were divided into amplicon sequence variants (ASVs) using the dada2 package v.1.14.1 [27]. Taxonomy was assigned to ASVs using the dada2 package with a 16S rRNA gene sequence database decorated with a nomenclature of GTDB r220 (https://zenodo.org/records/13984843 (accessed on 27 October 2025)). Alpha diversity metrics were calculated using the phyloseq package v.1.30.0 [28]. The prokaryotic phyla co-occurrence network was constructed using the phylosmith package v.1.0.7 with a rho threshold of 0.4 and a *p*-value threshold of 0.05 (https://joss.theoj.org/papers/10.21105/joss.01442 (accessed on 27 October 2025)), and was visualized using the ggrpah package v.2.2.1 (https://ggraph.data-imaginist.com (accessed on 27 October 2025)). Pairwise Spearman’s rank correlations between the relative abundance of different prokaryotic classes and environmental variables were calculated in the “microbiome” package v.1.8.0 (https://bioconductor.org/packages/release/bioc/html/microbiome.html (accessed on 27 October 2025)). To estimate beta diversity, a non-metric multidimensional scaling (NMDS) ordination with Bray–Curtis dissimilarity matrix was performed using the vegan v.2.6-4 package (https://CRAN.R-project.org/package=vegan (accessed on 27 October 2025)).

## 3. Results

### 3.1. Characteristics of the Sampling Sites

During the expedition to the BRZ in August 2024, twenty-eight samples of water, sediments, microbial mats, and biofilms were collected from hot springs of seven thermal fields (Umkhei, Kuchiger, Garga, Uro, Gusikha, Goryachinsk, Zmeiny) located in active or abandoned balneological resorts and natural environment (Figure 1, Table 1, and Supplementary Materials S1).

On the Umkhei thermal field, sediment samples were collected from two hot springs—4703 (T 32 °C, pH 7.6) and 4704 (T 42 °C, pH 9.5). Such differences in temperature and pH most likely indicated varying degrees of mixing between deep thermal and surface meteoric waters. The thermal waters of both springs were characterized by negative Eh values (Table 1 and Supplementary Materials S1).

On the Kuchiger thermal field, bottom sediments (#4705) were sampled from a site of natural deep thermal water discharge at a depth of 1.5 m. The temperature of the water at the surface of the thermal spring was 41 °C, pH was 6.9. Unfortunately, we were unable to measure the temperature and pH in the bottom sediments, but according to the literature data, the temperature can vary in the range from 40 °C to 57 °C, and the pH in the range from 9.0 to 10.2 [1,5,16,18,29].

The Garga hot spring is located in the abandoned balneological resort in the valley of the Garga River, right bank. Samples #4707-4711 were close for their parameters (72–74 °C, pH 7.8–7.9, Eh −160 mV and −190 mV), while sample #4715 was distinct (59 °C, pH 8.2, Eh +5 mV) (Table 1 and Supplementary Materials S1).

On the Uro thermal field located within the Uro River basin, all samples (#4718-4725) were characterized by temperature range 42–59 °C and close pH values 8.8–9.0 (Table 1 and Supplementary Materials S1), but they differed in their redox potential, which gradually decreased from positive values (Eh +20 mV in the sample #4719) to negative (Eh −200 mV in the sample #4725).

Samples #4728 (53 °C, pH 8.3, Eh +55 mV) and #4730 (51 °C, pH 9.1, Eh −115 mV) were collected in Gusikha hot springs located at the foot of the Ikatsky Range on the right bank of the Malaya Gusikha River (Table 1 and Supplementary Materials S1).

The Goryachinsk thermal field (samples #4731-4735) is located in Goryachinsk Village. The temperature range in the sampling sites was 45–52 °C, pH range was 7.2–9.1 (Table 1 and Supplementary Materials S1). The sample #4732 was characterized by a positive redox potential value (Eh +10 mV), while other samples were of the negative Eh range from −115 mV to −300 mV (Table 1 and Supplementary Materials S1).

The sample #4736 was collected from sediments of the Zmeiny hot spring (Svyatoy Nos Peninsula, Zmeinaya Bay) and was characterized by a temperature of 41 °C, pH 9.3, and Eh −350 mV.

Water types and physicochemical parameters for all springs are provided in Supplementary Materials S2.

### 3.2. Alpha and Beta Diversity of Microbial Communities of the BRZ Hot Springs

**Figure 2:**
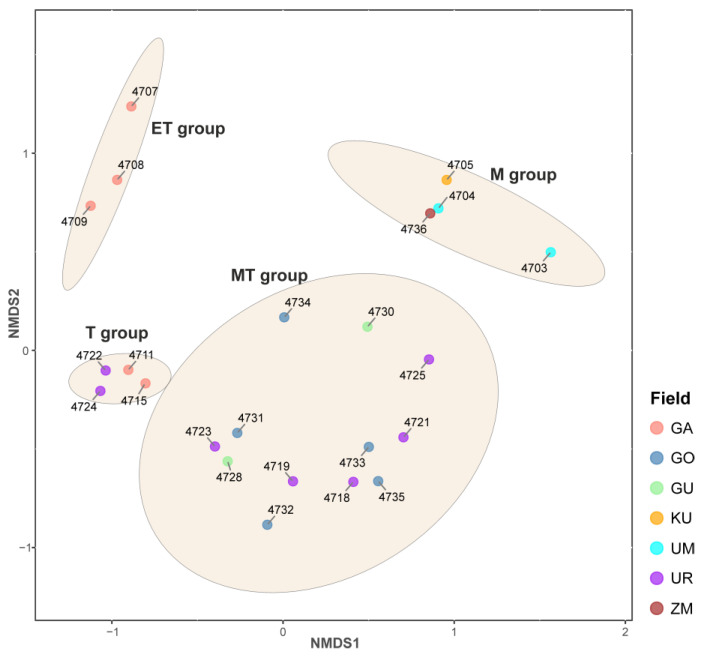
Analysis of beta diversity of microbial communities (ASVs which make up ≥0.2% of microbial community of at least one sample) via NMDS ordination based on Bray–Curtis distances (k  =  3; stress value  =  0.098). Thermal fields: GA—Garga, GO—Goryachinsk, GU—Gusikha, KU—Kuchiger, UM—Umkhei, UR—Uro, ZM—Zmeiny. Microbial community groups: ET—Extreme Thermophilic, T—Thermophilic, MT—Moderate Thermophilic, and M—Mixed.

Microbial communities of the BRZ hot springs were analyzed by high-throughput sequencing of 16S rRNA gene fragments. Following the application of all quality filters, the 23 samples analyzed in this study yielded between ~10,000 and ~35,000 sequences per sample. Alpha diversity analysis revealed a huge variety of Shannon (1.58–5.19), inverse Simpson indices (2.33–76.78), and Chao1 richness estimator (23–515) (Table 1). NMDS analysis of the microbial abundance profiles revealed weak clustering of microbial communities of the BRZ hot springs that was dependent on temperature rather than on the spring’s affiliation with any thermal field (Figure 2). All communities were allocated to four groups linked with the temperature range of sampling sites (Figure 2): the Extreme Thermophilic group (ET group), the Thermophilic group (T group), the Moderate Thermophilic group (MT group), and the Mixed group (M group). Since the pH range of the collected samples showed little variation, it was likely not a driving factor behind this clustering.

#### 3.2.1. Extreme Thermophilic Group

**Figure 3:**
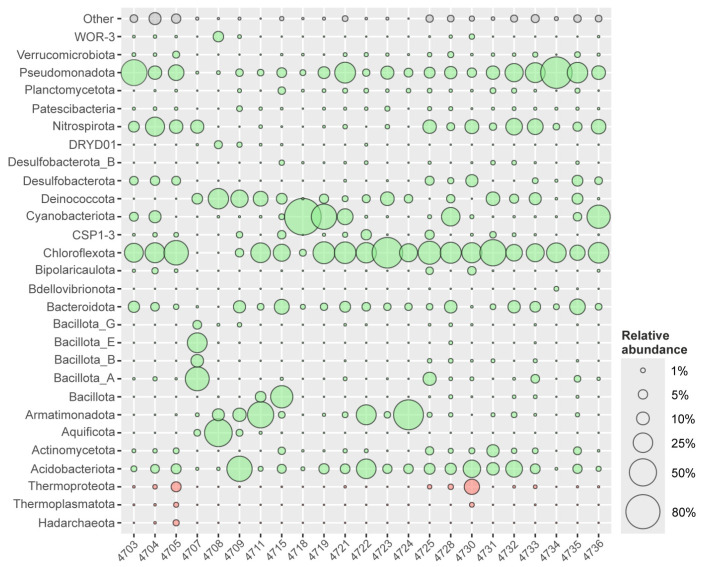
Taxonomic composition of microbiomes inhabiting the studied Baikal Rift Zone hot springs at the phyla level (only phyla, whose abundance was higher than 1% at least in one sample, are shown). Red and green bubbles indicate phyla of *Archaea* and *Bacteria* domains, respectively, while the gray bubble indicates sequences assigned to phyla comprising less than 1% of the community in all samples or sequences not assigned to any phyla.

The ET group included three microbial communities (#4707, #4708, #4709) of the Garga hot spring that were collected in the area of the thermal water outlet with a temperature of 74 °C. These microbial communities differed significantly from all other studied communities of the BRZ and were characterized by extremely low alpha diversity (Table 1, Figure 2 and Figure 3). ASVs affiliated with genus *Thermus* (*Deinococcota*), uncultivated representatives of the family *Aquificaceae* named UBA11096 (*Aquificota*), unclassified genus HR10 within the class *Blastocatellia* (*Acidobacteriota*), uncultured bacteria of the order *Fimbriimonadales* named LDYB01 (*Armatimonadota*), as well as uncultivated phyla DRYD01 and WOR-3 were detected in all three microbial communities. However, these three communities also differed significantly from each other.

In microbial communities of the sample #4707, the most abundant phylum was *Bacillota*_A (38.4% of all prokaryotes), represented by genera *Thermovenabulum* (23.8%), *Caloramator* (12.6%), and *Caldicellulosiruptor* (2.0%). The second dominant component was uncultured bacteria of the class-level phylogenetic lineage JACIYH01 (25.9%) belonging to the phylum *Bacillota*_E. Relatively abundant groups were representatives of the families *Desulfovirgulaceae* (4.9%) and *Moorellaceae* (4.6%) of the phylum *Bacillota*_B (9.7%), as well as of the order *Limnochordales* (4.4%) belonged to the phylum *Bacillota*_G. Representatives of the genera *Thermodesulfovibrio* (*Nitrospirota*) and *Thermus* (*Deinococcota*) accounted for 10.7% and 6.8% of all prokaryotes in the studied microbial community, respectively. The share of unclassified bacteria UBA11096, belonging to the family *Aquificaceae* (*Aquificota*), amounted to 2.4%.

The microbial community of Garga hot spring #4708 included only five dominant components. The most abundant groups were genera UBA11096 (*Aquificota*) and *Thermus* (*Deinococcota*) that comprised 52.1% and 27.6% of all prokaryotes, respectively. The subdominant bacteria in the studied community were uncultivated representatives of the phyla *Armatimonadota* (8.8%), WOR-3 (6.5%), and DRYD01 (3.6%). The dominant group of microbial community of dark gray deposits on wood (sample #4709) was the uncultivated genus HR10 of the class *Blastocatellia* (*Acidobacteriota*) that comprised 43.2% of all prokaryotes. The next most abundant prokaryotes belonged to the phyla *Deinococcota* (19.5%) represented by the single genus *Thermus*, *Armatimonadota* (11.0%) represented by unclassified genus LDYB01 of the order *Fimbriimonadales* (10.8%), as well as *Bacteroidota* (9.1%) represented by genus *Rhodothermus* (2.8%) and uncultured order-level phylogenetic lineage JANXDC01 (5.9%). Other significant phyla were *Chloroflexota* (4.0%), *Pseudomonadota* (3.1%), *Aquificota* (2.5%), CSP1-3 (2.1%), *Patescibacteria* (1.6), and DRYD01 (1.3%).

#### 3.2.2. Thermophilic Group

Two microbial communities of the Garga thermal field (#4711, #4715) and two microbial communities of the Uro thermal field (#4722, #4724) collected in hot springs with temperatures from 58 to 72 °C had low taxonomic diversity (Table 1) and fell into T group, which was characterized by the presence of ASVs affiliated with uncultivated genera GBS-DC (0.4–54.9%) and LDYB01 (1.7–20.5%) of the order *Fimbriimonadales* within the phylum *Armatimonadota* (2.4–61.0%), as well as genera *Chloroflexus* (3.4–11.5%), *Kallotenue* (2.5–15.7%) and *Caldilinea* (0.7–4.8%) of the phylum *Chloroflexota* (18.7–27.2%). In addition, the simultaneous presence of two genera of the phylum *Deinococcota*, *Thermus* (1.1–9.3%) and *Meiothermus* (1.0–5.3%), was shown for all microbial communities of this group.

The microbial community of the Garga thermal field (sample #4711) was characterized by the dominance of the genera GBS-DC (44.0%) and LDYB01 (1.8%) of the phylum *Armatimonadota* (46.4%), genera *Chloroflexus* (10.0%), *Roseiflexus* (4.6%), *Kallotenue* (8.6%), and *Thermanaerothrix* (1.4%) of the phylum *Chloroflexota* (25.6%), as well as genera *Thermus* (9.3%) and *Meiothermus* (4.5%) of the phylum *Deinococcota* (13.8%) was shown.

The microbial community of the Garga thermal field (sample #4715) included the same taxonomic groups, but in different proportions. The dominant components were the phylum *Bacillota* (33.1%), among which genera *Sutcliffiella*_A (27.8%) and *Alkalihalophilus* (2.3%) prevailed, as well as the phylum *Chloroflexota* (18.7%) represented by genera *Chloroflexus* (7.4%), *Kallotenue* (2.5%), *Caldilinea* (4.8%), and uncultivated bacteria of the class *Dehalococcoidia* (3.5%). Among *Bacteroidota* (14.1%), the most abundant group belonged to the genus *Rhodothermus* (8.4%), while among *Deinococcota* (6.9%), the genera *Meiothermus* (5.3%) and *Thermus* (1.6%) dominated. The phylum *Pseudomonadota* (5.6%) was represented mainly by unclassified bacteria of the family *Geminicoccaceae* (4.2%). Other significant phyla were *Acidobacteriota* (4.6%), CSP1-3 (4.0%), *Planctomycetota* (3.0%), *Actinomycetota* (2.9%), *Armatimonadota* (2.4%), *Cyanobacteriota* (1.7%), and *Desulfobacterota*_B (1.4%).

Microbial community of the Uro thermal field (sample #4722) was represented by the phyla *Chloroflexota* (27.2%), *Armatimonadota* (26.3%), *Acidobacteriota* (25.4%), CSP1-3 (6.3%), *Bacteroidota* (4.2%), *Deinococcota* (3.6%), and *Pseudomonadota* (2.1%). Among *Chloroflexota*, the most abundant groups belonged to the genera *Chloroflexus* (11.5%), *Kallotenue* (9.2%), and *Caldilinea* (4.8%), while the phylum *Armatimonadota* was represented by uncultured genera LDYB01 (20.5%) and GBS-DC (5.7%). The main taxa of the phylum *Acidobacteriota* were the unclassified representatives of the families HR10 (13.2%) and *Pyrinomonadaceae* (11.8%). The main components of the microbial community of sample #4724 were the phylum *Armatimonadota* (61.0%), represented by unclassified genera GBS-DC (54.9%) and LDYB01 (5.9%), and the phylum *Chloroflexota*, represented by the genera *Chloroflexus* (15.7%), *Kallotenue* (3.4%), and *Caldilinea* (2.1%). A subdominant part of the community was represented by the phyla *Acidobacteriota* (5.3%), *Pseudomonadota* (4.4%), *Deinococcota* (4.0%), and *Bacteroidota* (3.0%).

#### 3.2.3. Moderate Thermophilic Group

The MT group consisted of 12 microbial communities (#4718, #4719, #4721, #4723, #4725, #4728, #4730–4735) of hot springs with moderately high temperatures of 42–59 °C from Uro, Gusikha, and Goryachinsk thermal fields. The microbial communities of this group differed significantly from each other in alpha diversity (Table 1, Figure 2 and Figure 3), which was usually associated with the specific sampling point in the multi-layered biogeocenosis. Phototrophic microbial mats characterized by low taxonomic diversity developed on the surface, whereas the heterotrophic microbial community of sediments located beneath the mats was characterized by higher alpha diversity.

The Uro thermal field is covered with structured, multi-layered, and multi-colors microbial mats overlying gray sediments. Microbial community of the most represented yellow-orange mats (sample #4719) predominantly consisted of the representatives of the phylum *Cyanobacteriota* (43.3%)—the genera *Thermoleptolyngbya* (20.3%), *Thermostichus* (17.9%), and *Gloeomargarita* (2.2%), as well as the genus *Roseiflexus* (23.4%) of the phylum *Chloroflexota* (32.0%). In addition, the genera *Thermaurantiacus* (1.7%), *Roseococcus* (1.3%), unclassified representatives of the families *Rhodobacteraceae* (1.6%) and *Xanthobacteraceae* (1.5%) of the phylum *Pseudomonadota* (8.5%) were identified. *Deinococcota* were represented only by the genus *Meiothermus* (4.9%). Relatively abundant groups were uncultured bacteria of the family *Bryobacteraceae* (6.0%) of the phylum *Acidobacteriota* (6.2%) and unclassified representatives of the order *Cytophagales* (1.5%) of the phylum *Bacteroidota* (2.9%). The community of dark green mats (sample #4718) was represented predominantly by uncultured *Cyanobacteriales* (78.4%) and the genus *Oscillatoria* (12.9%) of the phylum *Cyanobacteriota* (93.0%). The dominant components of pink mats (sample #4723) were *Roseiflexus* (*Chloroflexota*) and *Meiothermus* (*Deinococcota*) comprised 60.5% and 11.7% of all prokaryotes, respectively. Among *Pseudomonadota* (10.6%), the most abundant groups belonged to uncultured taxa of the orders *Xanthomonadales* (6.3%) and *Geminicoccales* (2.0%). An intermediate slimy white layer transitioning into grayish sediments (sample #4721) was located under the phototrophic mats. A significant decrease in phototrophic bacteria was observed in the microbial community of this intermediate layer; in particular, the share of the genera *Thermoleptolyngbya* and *Thermostichus* reduced up to 5.0% and 2.4%, respectively. Overall, the proportion of *Cyanobacteriota* was 16.1% of all prokaryotes. The most abundant phylum was *Chloroflexota* (31.1%), primarily due to uncultured chemotrophic representatives of the class *Anaerolineae* (21.0%), rather than the phototrophic genus *Roseiflexus* (8.8%). The second dominant component was the phylum *Pseudomonadota* (28.7%) represented by diverse bacteria of the orders *Sphingomonadales* (8.8%), *Acetobacterales* (3.5%), *Geminicoccales* (3.0%), *Burkholderiales* (3.8%), and some others. In addition, uncultivated representatives of the order *Bryobacterales* (5.2%) of the phylum *Acidobacteriota* (5.5%) and the orders *Cytophagales* (3.1%) and *Kapaibacteriales* (2.0%) of the phylum *Bacteroidota* (7.1%) were quite abundant. A subdominant part of the community was represented by the phyla *Deinococcota* (1.8%), *Armatimonadota* (1.7%), CSP1-3 (1.2%), and *Planctomycetota* (1.1%). The microbial community of the underlying gray sediments (sample #4725) was characterized by the dominance of diverse uncultured representatives of the classes *Anaerolineae* (33.4%) and *Dehalococcoidia* (2.0%) of the phylum *Chloroflexota*. A distinctive feature of this microbial community was the relatively high content of uncultured bacteria of the families *Dissulfurispiraceae* (4.8%) and SM23-35 (6.5%) belonging to the phylum *Nitrospirota* (11.4%). The next most abundant groups were the phylum *Bacillota*_A (10.7%), mainly represented by the genus *Caloramator* (8.4%), the phylum *Acidobacteriota* (7.1%) represented by unclassified bacteria of the orders *Bryobacterales* (4.0%) and *Aminicenantales* (2.7%), as well as the phylum *Pseudomonadota* (7.1%) represented by diverse bacteria of the orders *Burkholderiales* (4.0%), *Rhizobiales* (1.6%), and others. Minor components of microbial community from the gray sediments were representatives of the phyla CSP1-3 (4.9%), *Desulfobacterota* (4.6%), *Actinomycetota* (3.8%), *Bipolaricaulota* (3.1%), *Bacteroidota* (2.2%), and *Armatimonadota* (1.2%).

The microbial community from a hot puddle (sample #4728) of the Gusikha thermal field was represented by phototrophic bacteria belonging to the genus *Fischerella* (21.3%) of the phylum *Cyanobacteriota* (22.5%), as well as to the genera *Chloroflexus* (6.5%) and *Roseiflexus* (1.1%) of the phylum *Chloroflexota* (29.4%) (Figure 3). Among *Chloroflexota*, the genera *Anaerolinea* (8.0%), *Bellilinea* (1.6%), *Kallotenue* (1.9%), and unclassified bacteria of the families UBA4823 (3.0%) and UBA3254 (2.0%) were also detected. The next most abundant phylum was *Bacteroidota* (9.6%), which was represented mainly by the uncultured class named UBA10030 (7.3%) and also class *Bacteroidia* (1.6%). The phylum *Pseudomonadota* (9.4%) mainly included the unclassified representatives of the orders *Burkholderiales* (4.4%), *Geminicoccales* (2.4%), and *Xanthomonadales* (1.0%), while the phylum *Acidobacteriota* (7.7%) was represented by the genus *Saccharicenans* (3.4%) and uncultured bacteria of the family *Bryobacteraceae* (3.9%). *Meiothermus* (3.9%) was the only identified genus in the phylum *Deinococcota*, whereas representatives of the order *Thermodesulfovibrionales* (3.2%) were the main group of the phylum *Nitrospirota* (3.4%). Moreover, diverse bacteria of the phyla *Desulfobacterota* (2.0%), *Verrucomicrobiota* (1.8%), and *Actinomycetota* (1.2%) were identified. The 16S rRNA gene profiling showed the predominance of the phyla *Chloroflexota* (26.8% of all prokaryotes) and *Acidobacteriota* (19.9%) in the microbial community of hot spring #4730. Among *Chloroflexota*, the most abundant groups belonged to diverse, mainly uncultured taxa of the classes *Anaerolineae* (13.2%) and *Dehalococcoidia* (6.5%), while the phylum *Acidobacteriota* (19.9%) was represented by the unclassified genus named JARYMI01 (18.1%) of the class *Aminicenantales*. The phylum *Nitrospirota* (12.1%) mainly included the genus *Dissulfurispira* (7.7%) and an uncultured family named SM23-35 (3.2%) of the order *Thermodesulfovibrionales* (11.8%). The main group of the phylum *Desulfobacterota* (9.1%) was the unclassified class BSN033 (7.5%).

The microbial community of dark green mat (sample #4734) of the Goryachinsk thermal field was represented by unclassified bacteria the family *Thiobacillaceae* (65.7%) of the phylum *Pseudomonadota* (67.9%) and the genera *Chloroflexus* (20.2%) of the phylum *Chloroflexota* (25.4%) (Figure 3). The microbial community in the sandy and stony sediments underlying the dark green mats (sample #4731) was dominated by the following phyla—*Chloroflexota* (46.6%), *Deinococcota* (11.6%), *Pseudomonadota* (11.1%), *Acidobacteriota* (9.6%), and *Actinomycetota* (9.2%) (Figure 3). Among *Chloroflexota*, the most abundant group belonged to the class *Anaerolineae* (41.0%) represented by diverse mostly uncultivated bacteria of the orders *Anaerolineales* (29.6%), *Aggregatilineales* (5.5%), J102 (4.0%), and *Caldilineales* (1.8%). In addition, the genus *Chloroflexus* (5.2%) was detected, apparently originating from the overlying dark green mat. The phylum *Deinococcota* was represented by the only genus *Meiothermus* (11.6%). The genus *Tepidimonas* (3.4%) was the most abundant group of the phylum *Pseudomonadota* that also included diverse bacteria of the orders *Burkholderiales* (6.4%), *Geminicoccales* (4.0%), and others. The phylum *Acidobacteriota* was represented by uncultivated bacteria of the classes *Blastocatellia* (4.9%) and *Terriglobia* (4.7%), while *Actinomycetota* consisted of an uncultured class named UBA4738 (8.1%).

The most abundant group of the microbial community of the orange-brown sandy sediments (samples #4732 and #4733) was the phylum *Pseudomonadota* (21.4 and 26.0%), represented by diverse bacteria of the families *Thiobacillaceae* (8.0 and 16.3%), *Burkholderiaceae* (1.8 and 2.2%), *Methylococcaceae* (1.3% only in #4732), *Geminicoccaceae* (4.6% only in #4732), *Xanthobacteraceae* (1.3 and 0.8%), and others. The second-most abundant group in #4732 was the phylum *Acidobacteriota* (18.1%), mainly included unclassified representatives of the orders *Bryobacterales* (8.5%), *Acidoferrales* (5.6%), and HR10 (2.8%), while in #4733, *Acidobacteriota* was only 5.7%. The phylum *Nitrospirota* (17.9% and 16.1%) was represented by unclassified bacteria of the family *Nitrospiraceae* in #4732 and by the genus *Dissulfurispira* (5.9%) in #4732, while the phylum *Chloroflexota* (17.7% and 21.1%) included diverse mostly uncultivated bacteria of the order *Anaerolineales* (10.7% and 9.8%). Unclassified bacteria of the families *Cytophagaceae* (6.5%) and *Saprospiraceae* (1.7%) belonged to the phylum *Bacteroidota* (9.4%) and were detected in #4732. The genus *Meiothermus* (*Deinococcota*) was also quite abundant and comprised 4.8% and 8.9% of all prokaryotes in microbial communities of #4732 and #4733, respectively. The minor components of #4732 community were the phyla *Armatimonadota* (1.6%), *Actinomycetota* (1.4%), *Planctomycetota* (1.2%), and some others. While the subdominant phyla in the microbial community of sample #4733 were *Bacteroidota* (6.9%), *Acidobacteriota* (5.7%), *Bacillota_A* (4.2%), *Desulfobacterota* (1.7%), *Actinomycetota* (1.6%), *Verrucomicrobiota* (1.2%), and some others.

The microbial community of black silt (sample #4735) was the most diverse of all studied communities. The most abundant group was the phylum *Pseudomonadota* (27.9%), which was represented by the families *Thiobacillaceae* (9.8%), *Burkholderiaceae* (2.6%), *Rhodocyclaceae* (2.1%), *Xanthobacteraceae* (1.4%), *Rhodobacteraceae* (1.4%), *Sphingomonadaceae* (1.3%), *Hyphomonadaceae* (1.1%), and others. The phylum *Bacteroidota* (15.6%) mainly included representatives of the families *Cyclobacteriaceae* (4.8%), *Saprospiraceae* (2.0%), F082 (1.5%), PHOS-HE28 (1.4%), *Ignavibacteriaceae* (1.2%), *Melioribacteraceae* (1.2%), while the phylum *Chloroflexota* (14.9%) was represented by the families *Villigracilaceae* (4.9%), *Anaerolineaceae* (1.8%), *Brachytrichaceae* (1.9%), *Phototrophicaceae* (1.8%), *Caldilineaceae* (1.0%), and some others. As in most other microbial communities of the BRZ hot springs, a high proportion of the genus *Meiothermus* (*Deinococcota*) was detected and comprised 7.2% of all prokaryotes. Quite an abundant group consisted of bacteria from the orders *Syntrophobacterales* (3.5%) and *Desulfobacterales* (2.7%) of the phylum *Desulfobacterota* (7.0%). In addition, significant numbers of the phyla *Acidobacteriota* (4.9%), *Nitrospirota* (4.8%), *Cyanobacteriota* (4.1%), *Actinomycetota* (3.4%), *Bacillota_*A (2.3%), *Verrucomicrobiota* (1.6%), and *Planctomycetota* (1.2%) were identified.

#### 3.2.4. Mixed Group

The M group comprised microbial communities from Umkhei (#4703 and #4704), Kuchiger (#4705), and Zmeinaya Bay (#4736) thermal springs, with the lowest temperature ranging from 32 to 42 °C and a significant anthropogenic effect. These microbial communities were characterized by quite high taxonomic diversity and the presence of ASVs affiliated with both thermophilic taxa described above for the MT group and with taxa represented by mesophilic organisms, including those introduced by humans.

Despite differences in temperature and pH values, microbial communities from both samples of Umkhei hot springs were similar to each other. The 16S rRNA gene profiling showed predominance of the phyla *Chloroflexota* (23.4% and 26.3% of all prokaryotes), *Pseudomonadota* (45.6% and 11.2%), and *Nitrospirota* (7.0% and 23.9%) in the microbial community of hot springs #4703 and #4704 (Figure 3). Among *Chloroflexota*, the most abundant groups belonged to diverse uncultured taxa of the classes *Anaerolineae* (20.8% and 14.3% of all prokaryotes), *Chloroflexia* (1.9% and 9.5%), and *Dehalococcoidia* (0.4% and 2.4%), while the phylum *Nitrospirota* was represented by uncultured bacteria of the order *Thermodesulfovibrionales*, mainly of the families *Dissulfurispiraceae* (4.4% and 17.3%) and SM23-35 (2.5% and 6.4%). The most significant difference in the microbial communities of #4703 and #4704 was a higher share of *Pseudomonadota* and a lower share of *Nitrospirota* in #4703. Among *Pseudomonadota*, the most abundant group belonged to the class *Gammaproteobacteria* (43.7% and 10.4%) represented mainly by the genus *Aeromonas* (10.0% and 2.5%) and uncultivated representatives of the family *Rhodocyclaceae* (7.7% and 2.5%) as well as the microbiome of the #4703 was characterized by the presence of sulfur-oxidizing bacteria of the genera *Thiocapsa* (8.2%), *Lamprobacter* (3.0%), *Thermochromatium* (1.6%), *Thiobacillus* (1.2%), *Thioflexithrix* (1.9%), and *Thiothrix* (1.6%). The subdominant phyla in the studied community were *Cyanobacteriota* (4.5% and 8.9%), *Desulfobacterota* (4.2% and 5.1%), *Acidobacteriota* (1.7% and 4.3%), *Bacteroidota* (7.7% and 4.2%), and *Bipolaricaulota* (2.0%—only in #4704, former “*Candidatus* Acetothermia” and OP1 division). The phylum *Cyanobacteriota* was represented by the genera *Pseudanabaena*, *Caldora*, *Phormidium*, *Koinonema*, *Leptodesmis,* and *Kamptonema* in #4703 and only the phototrophic genera *Koinonema* and *Leptodesmis* in #4704. The main taxon of the phylum *Desulfobacterota* was the uncultured class BSN033 (1.2% and 3.8%). *Archaea* were a minor component of the microbial community #4704, comprising only 1.7% of all prokaryotes.

Similar to the microbial communities of the Umkhei thermal field, the dominant component of the microbial community of hot spring #4705 (Kuchiger thermal field) was the phylum *Chloroflexota* (40.3%), among which uncultured taxa of the classes *Anaerolineae* (18.7%) and *Dehalococcoidia* (21.3%) prevailed (Figure 3). Interestingly, 18.1% of the all sequences were related to the order-level group GIF9 of the class *Dehalococcoidia*. Moreover, the community of #4705 spring was also characterized by the dominance of the phylum *Nitrospirota* (11.3%), including uncultured bacteria of the families *Dissulfurispiraceae* (4.8%) and SM23-35 (6.5%), as well as the phylum *Desulfobacterota* (4.5%), including the uncultured class BSN033 (4.0%). Simultaneously, the microbial community of #4705 spring exhibited several distinct differences. First, the phylum *Pseudomonadota* (15.8%) was represented mainly by the family *Enterobacteriaceae* (15.4%), primarily by representatives of the genera *Enterobacter* (6.5%) and *Serratia* (6.1%), which indicates a significant anthropogenic effect on the Kuchiger thermal field. Second, phototrophic taxa were absent, presumably because of the sampling depth. Third, the phylum *Acidobacteriota* (5.9%) was represented by uncultivated bacteria of the classes *Aminicenantia* (2.4%) and UBA6911 (2.8%), while the phylum *Bacteroidota* (1.4%) included uncultured bacteria of the class UBA10030 (1.1%). Fourth, a significant number of the phyla *Verrucomicrobiota* and *Actinomycetota* representatives were identified, the share of which comprised 2.6% and 1.7% of all prokaryotes, respectively. Fifth, archaea constituted 9.5% of all prokaryotes and were represented mainly by uncultured representatives of the phyla *Thermoproteota* (5.9%), *Hadarchaeota* (2.0%), and *Thermoplasmatota* (1.2%).

The microbial community of sample #4736 was represented by phototrophic bacteria belonging to the genus *Leptodesmis* (35.8%) of the phylum *Cyanobacteriota* (36.1%) and the family *Chloroflexaceae* (21.5%) of the phylum *Chloroflexota* (28.1%) (Figure 3). Among *Chloroflexota*, diverse heterotrophic bacteria of the class *Anaerolineae* (6.3%) were also detected. The phylum *Nitrospirota* (13.2%) was mainly represented by uncultivated bacteria of the family *Dissulfurispiraceae* (11.3%), while the phylum *Pseudomonadota* (11.7%) included the families *Rhodocyclaceae* (5.1%), *Chromatiaceae* (4.9%), and others. The subdominant phyla in the microbial community of sample #4736 were *Desulfobacterota* (3.2%) and *Bacteroidota* (2.4%), as well as some others with a share of less than 1%.

### 3.3. Co-Occurrence Network Analysis

**Figure 4:**
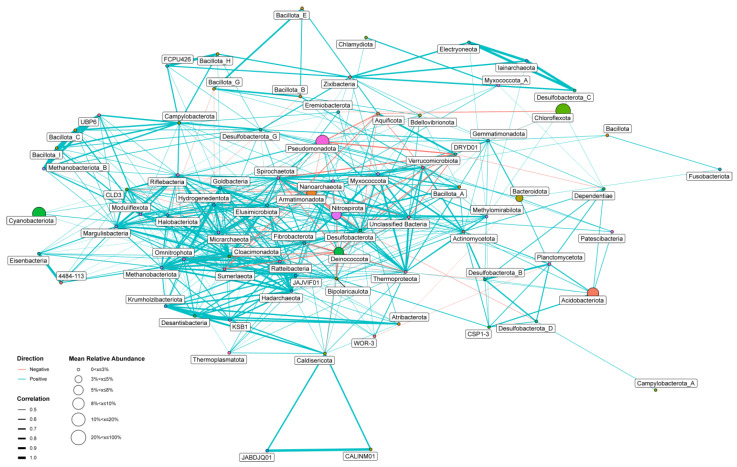
Co-occurrence network of microbial communities of the Baikal Rift Zone hot springs at the phyla level.

The co-occurrence network analysis (Figure 4) revealed 77 phyla, the presence of which was moderately or strongly correlated (≥0.4 or ≤−0.4) with the presence of other phyla. Among them, 42 phyla with ≥10 edges were observed. The phyla were distributed between the two groups. The first small group included *Armatimonadota*, *Deinococcota*, *Aquificota*, and DRYD01, which were integral components of the microbial communities of the hot springs with the highest temperatures (58–74 °C), i.e., these were extremely thermophilic and thermophilic microbial communities with low biodiversity (see above). The second large group comprised the absolute majority of the remaining phyla, including *Pseudomonadota*, *Nitrospirota*, *Desulfobacterota*, *Actinomycetota*, *Verrucomicrobiota*, *Spirochaetota*, *Bacillota*_A, *Bipolaricaulota* (*Acetothermia*), *Bdellovibrionota*, *Thermoproteota*, *Hadarchaeota*, and others. Representatives of these taxa were part of more diverse moderately thermophilic and mixed microbial communities of thermal springs with lower temperatures from 32 to 59 °C. The hub-taxa within each group had positive correlations with each other, while the hub-taxa from different groups had negative correlations. Therefore, negative correlations in the main were observed for the phyla from the first small group, because the number of phyla in the first group was significantly lower than in the second group. In particular, the most numerous negative interactions were found for *Armatimonadota* and *Deinococcota,* with 17 edges for each, while the number of positive interactions for these phyla was four and three edges, respectively. On the contrary, the phyla from the second large group had predominantly positive interactions with other hub-taxa. The most frequent positive interactions were observed within the *Desulfobacterota* (25 edges), followed by the *Nitrospirota* (21 edges), *Pseudomonadota* (17 edges), *Actinomycetota* (19 edges), *Verrucomicrobiota* (16 edges), *Bacillota*_A (14 edges), and *Bdellovibrionota* (10 edges). Furthermore, certain uncultivated deep-branching bacterial lineages displayed significant co-occurrence, including *Bipolaricaulota* (*Acetothermia*) (24 edges). Among the *Archaea,* the *Thermoproteota* (22 edges) and *Hadarchaeota* (22 edges) demonstrated the most abundant positive interactions. Additionally, for representatives of the first small group, a positive correlation between abundances at the class level and temperature was found, while for representatives of the second large group, this correlation was negative (Figure S1 of the Supplementary Materials S2). Thus, the co-occurrence of hub-taxa is determined primarily by the influence of temperature, and negative links reflected the very different temperatures of the hot springs that prokaryotes inhabit. Interestingly, the phyla *Chloroflexota* and *Cyanobacteriota* were among the most abundant taxa in the studied microbial communities but had one of the lowest numbers of interactions. Probably low number of connections for *Chloroflexota* (two positive and three negative) with other hub-taxa could be explained by diversity of identified metabolic groups of representatives of this phylum (phototroph/chemotroph, lithotroph/organotroph, autotroph/heterotroph), which were detected in all type thermal springs, while low number of edges for *Cyanobacteriota* (four positive) indicates that presence of these primary producers might be relatively independent of other taxa.

### 3.4. Taxonomic Diversity of Microbial Communities of the Enrichment Cultures

**Figure 5:**
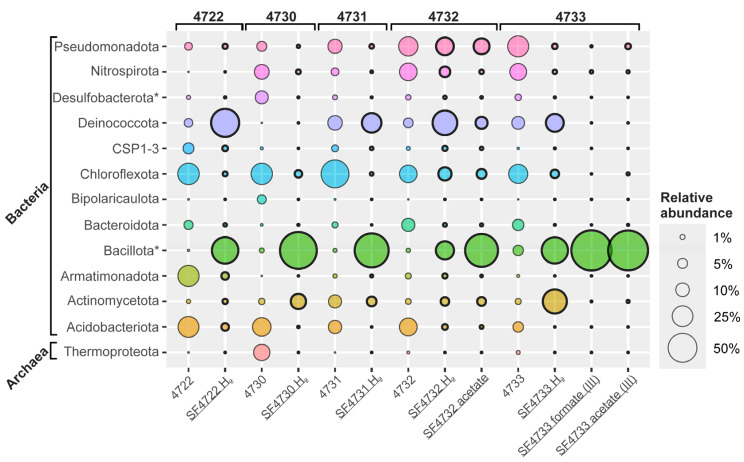
Comparison of the taxonomic composition of the initial environmental microbiomes and the enrichment cultures obtained on the media with synthesized ferrihydrite and non-fermentable electron donors at the phyla level. Only phyla with an abundance higher 1% in at least in one enrichment are shown. Different bubble colors indicate assignment to certain phyla. Enrichment cultures are underlined. * - in this figure, *Bacillota* row includes *Bacillota*, *Bacillota*_A, _B, _D, _E, _G, _H.

**Figure 6:**
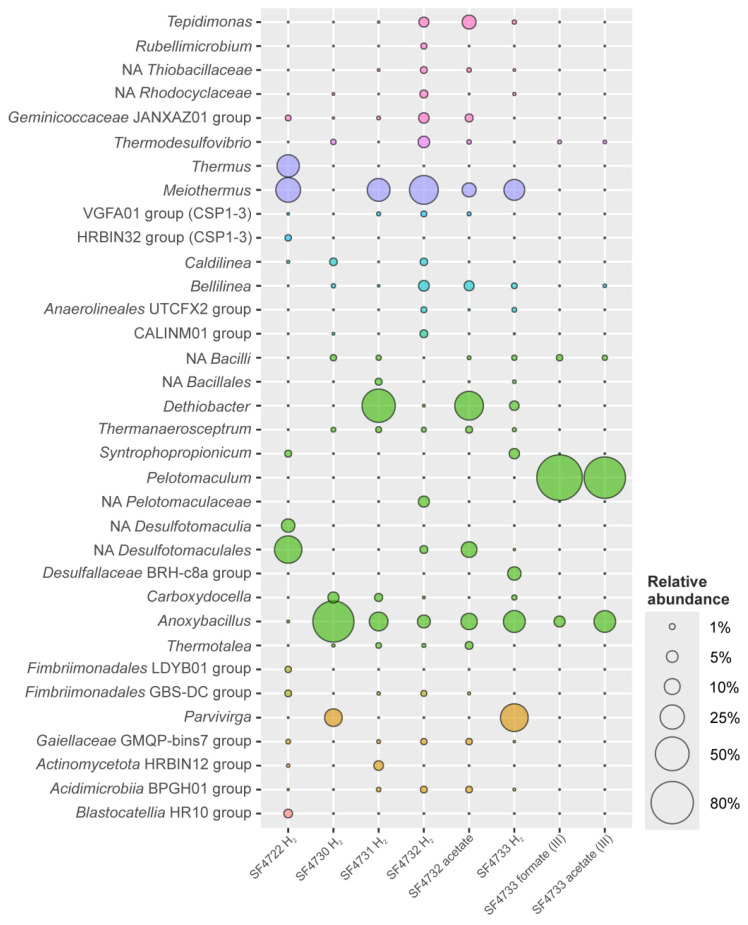
Taxonomic composition of the enrichment cultures microbiomes obtained on the media with synthesized ferrihydrite and non-fermentable electron donors at the genera level (only genera with an abundance higher than 1% in at least one enrichment are shown). Bubble color denotes the belonging of different generas to certain phyla (in this figure, *Bacillota* row includes *Bacillota*, *Bacillota*_A, _B, _D).

After three weeks of incubation of the samples on selective media, a reduction in ferrihydrite accompanied by the blackening of the sediment was observed in samples #4722 (Uro thermal field), #4730 (Gusikha thermal field) with hydrogen, and in all samples from the Goryachinsk thermal field—#4731 with hydrogen, #4732 with hydrogen or acetate, #4733 with hydrogen. The cultures were dominated by morphologically diverse rods (various lengths and thicknesses), most of which were attached to mineral particles. A small number of cocci have also been observed in the enrichment culture originating from sample #4733. Further, this culture was transferred for two times with formate (10 mM) or acetate (10 mM) as the electron donor and ferrihydrite as the electron acceptor. The 16S rRNA gene profiling revealed depletion of initial phyla diversity, with domination of phylotypes belonging to *Bacillota*, *Deinococcota,* or *Acidobacteriota* (Figure 5). On the family-genus level, the total diversity of the enrichment cultures was slightly decreased (Figure 6). At the same time, a few phylotypes that exhibited a marked increase in relative abundance gained a significant advantage compared to the natural samples (Figure 6).

#### 3.4.1. Microbial Community Structure of the Enrichment from Sample #4722 (Uro Thermal Field)

In general, the taxonomic diversity in the enrichment culture with molecular hydrogen and ferrihydrite was comparable to that of the natural sample. However, at the phylum level, enrichment culture showed the pronounced redistribution in relative abundance compared to the native sample (here and further, the first number refers to natural samples, the second number after the slash refers to the enrichments). Most of the community was composed of phyla *Deinococcota* (3.6/47.3%) and *Bacillota* (0.09/40.9%). The remaining phyla comprised only a small part of the community: *Chloroflexota* (27.2/0.09%), *Armatimonadota* (26.3/2.7%), *Acidobacteriota* (25.4/2.7%), CSP1-3 (6.3/1.3%), and *Pseudomonadota* (2.1/1.0%). At the genus level, a significant shift in the relative abundance of phylotypes was observed. Selective conditions provided the growth of specific bacterial lineages; the abundance of most phylotypes decreased compared to the natural sample. The most abundant phylotypes belonged to the genera *Meiothermus* (2.5/26.2%) and *Thermus* (1.0/21.2%). Two deep-branching uncultured representatives of the *Bacillota* phylum made up one-third of the community (relative abundance 32.5% and 6.9%). Notably, these two phylotypes were not detected (ND) in the native sample. Decreasing relative abundance was detected for *Chloroflexus* (11.5/<0.01%), *Kallotenue* (9.2/0.05%), *Caldilinea* (4.8/<0.01%), HR10 (13.2/2.5%), *Pyrinomonadaceae* (11.8/<0.01%), HRBIN32 (6.0/1.2%).

#### 3.4.2. Microbial Community Structure of the Enrichment from Sample #4730 (Gusikha Thermal Field)

In general, the taxonomic diversity of the enrichment culture with molecular hydrogen and ferrihydrite was decreased compared to that of a natural sample. *Archaea* were completely eliminated (16.1/0.01%), as well as bacterial phylum *Acidobacteriota* (19.9/<0.01%). The 16S rRNA gene profiling showed the predominance of the phyla *Bacillota* (0.7/82.3%) and *Actinomycetota* (1.8/12.8%), while phylotypes belonging to the phylum *Chloroflexota* depleted—2.6% compared to 26.8% in the native sample. Among *Bacillota*, the most abundant were two genera *Anoxybacillus* (<0.01/75.7%) and *Carboxydocella* (4.5%). This phylotype was not found in the native sample, as was the *Parvivirga* representative of the *Actinomycetota* phylum, which accounted for 12.5% of the enrichment.

#### 3.4.3. Microbial Community Structure of the Enrichment from Sample #4731 (Goryachinsk Thermal Field)

The taxonomic diversity of the enrichment culture with molecular hydrogen and ferrihydrite was decreased compared to the natural sample. At the phylum level, enrichment culture with molecular hydrogen and ferrihydrite was predominantly composed of *Bacillota* (0.52/70.3%) and *Deinococcota* (11.6/22.1%). Representation of the other phyla detected in the native sample decreased—*Chloroflexota* (46.6/0.4%), *Pseudomonadota* (11.1/0.75%), *Acidobacteriota* (9.6/ND), and *Actinomycetota* (9.2/4.8%). At the family-genus level, three phylotypes accounted for 84% of the total community. The genus *Dethiobacter* was the most abundant (48.7%). This phylotype was not found in the native sample, as was the *Anoxybacillus,* the second representative of the *Bacillota* phylum, which accounted for 14.6% of the enrichment. The relative abundance of the following phylotypes has noticeably changed in comparison to that of the native sample: *Meiothermus* (11.6/22%), uncultured class of *Actinomycetota* named UBA4738 (3.3/8.1%), *Carboxydocella* (ND/2.1%), *Chloroflexus* (5.2/ND), uncultivated bacteria of the orders *Anaerolineales* (29.6/ND), *Aggregatilineales* (5.5/ND), and J102 (4.0/ND).

#### 3.4.4. Microbial Community Structure of the Enrichment from Sample #4732 with Molecular Hydrogen and Ferrihydrite

This enrichment culture was characterized by a high diversity, both at the phylum and family-genus level, but with a pronounced shift in the dominant taxa compared to that of the native sample. Representatives of the *Deinococcota* (4.8/36.1%), *Bacillota* (0.69/18.8%), *Chloroflexota* (17.7/9.4%), along with *Pseudomonadota* (21.7/18.0%) and *Nitrospirota* (17.9/5.8%) comprised 67% of all phylotypes. *Meiothermus* (4.8/36.1%) and *Anoxybacillus* (0.3/6.4%) were the most abundant genera. The following phylotypes were not detected in the sample #4732 but appeared in the enrichment culture: *Thermodesulfovibrio* (ND/4.9%), *Bellilinea* (ND/4.2%), *Tepidimonas* (ND/4.2%), uncultured *Pelotomaculaceae* (ND/4.2%), uncultured *Rhodocyclaceae* (ND/2.1%), and uncultured *Desulfotomaculales* (ND/2.0%). It is also worth noting that 2% of this enrichment consisted of the new phylum CALINM01.

#### 3.4.5. Microbial Community Structure of the Enrichment from Sample #4732 with Acetate and Ferrihydrite

The microbial community of this enrichment culture exhibited a taxonomic composition similar to that of the enrichment with hydrogen. However, some phylotypes were selectively enriched, while the relative abundance of others decreased compared to their abundance in the native sample or the enrichment culture with hydrogen (first and second numbers after slash): *Dethiobacter* (ND/0.1/35.9%), uncultured *Desulfomaculales* (ND/2.0/9.8%); *Anoxybacillus* (0.3/6.4/10.8%), *Tepidimonas* (ND/4.2/7.5%); *Meiothermus* (4.8/36.1/7.7%). The taxonomic composition of the uncultured bacteria was similar to that of the enrichment with hydrogen added as a substrate.

#### 3.4.6. Microbial Community Structure of the Enrichment from Sample #4733 with Molecular Hydrogen and Ferrihydrite

The taxonomic diversity of this enrichment culture comprised *Bacillota* (5.52/41.2%) represented by the most abundant *Anoxybacillus* (<0.01/20.3%), uncultured *Desulfomaculales* BRH-c8a (ND/7.0%), *Syntrophopropionicum* (ND/3.9%), and *Dethiobacter* (ND/3.2%); *Deinococcota* (8.9/18.3%), represented by *Meiothermus*; *Actinomycetota* (1.6/34.3%), with the most abundant being *Parvivirga* (33.3%). *Pseudomonadota* (1.2%), *Chloroflexota* (3.5%), and *Nitrospirota* (0.7%), which were the most diverse and abundant phyla in the native sample, collectively comprised less than 5% of the enrichment culture.

#### 3.4.7. Microbial Community Structure of the Enrichment from Sample #4733, with Formate and Ferrihydrite (The Third Transfer)

**Figure 7:**
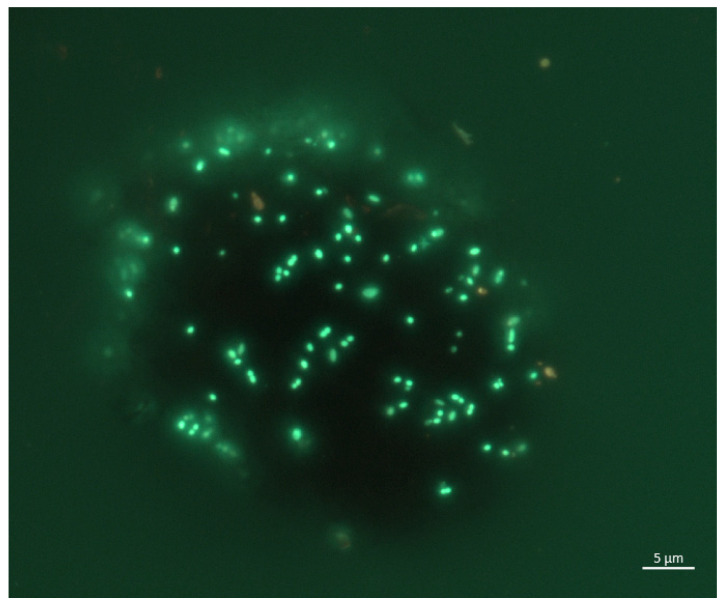
Cellular morphology of the dominant bacterium in the enrichment culture (third transfer) with ferrihydrite and formate from sample #4733. Cells stained by acridine orange.

The taxonomic diversity of this enrichment was represented only by four phylotypes—three representatives of the phylum *Bacillota*—*Pelotomaculum* (93.8%), *Anoxybacillus* (4.5%), NA *Bacilli* (1.2%), and *Thermodesulfovibrio* (0.3%) of *Nitrospirota*. The high dominance of *Pelotomaculum* in this enrichment culture (Figure 6) allowed us to identify its cell morphology. It is most probable that they were cocci located singly, in pairs, or in chains or clusters, closely associated with ferrihydrite particles (bright spots in Figure 7). This stable enrichment will be used in the future for further replanting and attempts to isolate a pure crop. BLAST analysis (ver. BLAST + 2.17.0) revealed low 16S rRNA gene identity of this bacterium with representatives of the genera *Thermoflavimicrobium* (91.9%), *Pelotomaculum* (91.6%), and *Neomoorella* (90.66%), as well as *Thermincola* (90.3%). All of these genera belong to different families, and such low sequence identity suggests that this bacterium may represent a novel taxon at a deep level within the phylum *Bacillota*.

#### 3.4.8. Microbial Community Structure of the Enrichment from Sample #4733, with Acetate and Ferrihydrite (Third Transfer)

The microbial community of this enrichment culture exhibited a taxonomic composition similar to that of formate, an uncultured representative of the *Bacillota* phylum (76.9%) and representative of the genus *Anoxybacillus* (19.8%) composed 96.7% of the four phylotypes.

## 4. Discussion

### 4.1. Microbial Communities of the BRZ

Microbial communities of the BRZ hot springs have been extensively studied previously, but these studies have typically focused on either phototrophic microbial mats or on the whole microbial community from one or a few sites [2,4,7,8,9,10,11,12,13,14,15,16]. In our work, we conducted the first large-scale screening of microbial communities from hot springs of the BRZ, located in Umkhei, Kuchiger, Garga, Uro, Gusikha, Goryachinsk, and Zmeiny thermal fields, using metabarcoding of the V3-V4 regions of 16S rRNA. The studied hot springs of the BRZ are polyextreme ecosystems characterized by a wide range of temperature (from 32 to 74 °C), predominantly alkaline pH values (up to 9.5), low salinity (up to 1 g/l), and various chemical compositions of water enriched with N_2_. Comparing the data from the beta diversity analysis and the temperature values in each sample, we noticed a strong dependence of the composition of the microbial community on temperature, what allowed us to classify microbial communities into four groups: Extreme Thermophilic (ET), Thermophilic (T), Moderate Thermophilic (MT), and Mixed (M). The ET group had the highest temperature at 74 °C, while the T group ranged from 58 to 72 °C. The MT group ranged between 42 and 59 °C, and the M group had low temperatures between 32 and 42 °C.

The ET group comprised three communities with low alpha diversity from the Garga hot spring. Despite differences, they shared key taxa, including *Thermus* (*Deinococcota*), the uncultivated genus UBA11096 (*Aquificaceae*, *Aquificota*), and some others. UBA11096, the dominant component of white fouling in spring #4708, likely functions as a primary producer of autochthonous organic matter. Previous culture-based studies of *Aquificota* suggested that these chemolithoautotrophic organisms typically oxidize hydrogen sulfide or hydrogen to generate energy, with oxygen serving as the terminal electron acceptor [30,31,32,33,34,35]. The generated organic matter is presumably consumed by aerobic heterotrophs like *Thermus* and potentially by the uncultured genus LDYB01 (*Fimbriimonadales*, *Armatimonadota*), consistent with the aerobic metabolism of cultivated *Armatimonadota*. Since cultivated representatives of *Thermus* and all cultivated representatives of the phylum *Armatimonadota* utilize a number of carbohydrates and proteinaceous substrates during aerobic respiration [36,37,38,39,40,41].

Two other microbial communities (#4707 and #4709) developed primarily on wood that fell into the Garga hot spring when a nearby wooden bathhouse collapsed. The presence of wood leads to the development of microbial communities that carry out the mineralization of organic matter. For example, the dominant groups of the sample #4709 were aerobic and facultative anaerobic heterotrophic bacteria of the uncultivated genus HR10 of the class *Blastocatellia* (*Acidobacteriota*), the genus *Thermus* (*Deinococcota*), unclassified genus LDYB01 (*Armatimonadota*), as well as *Bacteroidota* represented by genus *Rhodothermus* and uncultured order-level phylogenetic lineage JANXDC01.

The microbial community of the brown-pink microbial mat (#4707) was represented by obligate and facultative anaerobic heterotrophic bacteria (*Thermovenabulum* [42], *Caloramator* [43], *Caldicellulosiruptor* [44], and *Thermus* [36]) that can utilize a number of carbohydrates and proteinaceous substrates and potential sulfate-reducing bacteria (SRB) such as *Thermodesulfovibrio* [45,46,47], *Desulfofundulus* [48], and probably by unclassified representatives of the family *Desulfovirgulaceae* and the class-level phylogenetic lineage JACIYH01 [49]. SRB accounted for up to 42% of all prokaryotes, which is shown for the first time for microbial communities of the BRZ hot springs, and it is strikingly different from previously published data for the Garga hot spring [2,4,10]. The significant SRB abundance likely explains the low Eh values (−190 mV), which is a novel finding for the Garga spring.

The T group, comprising microbial communities with low alpha diversity from the Garga and Uro thermal fields, is distinguished from the ET group by a reduced abundance of *Aquificota*, DRYD01, and WOR-3, and an increased prevalence of *Armatimonadota* and *Deinococcota*. The phylum *Armatimonadota* included the genera LDYB01 and GBS-DC, the latter comprising probable aerobic heterotrophs [37,38,39,40,41]. The phylum *Deinococcota* was represented by *Thermus* and *Meiothermus*. Unlike *Thermus*, *Meiothermus* has a lower temperature optimum, enabling it to successfully compete with bacteria of the genus *Thermus* at decreased temperatures [36,50]. Both genera aerobically respire carbohydrates, amino acids, organic acids, and polyols, with some species capable of nitrate reduction [36,50]. The T group was further distinguished from the ET group by the presence of *Chloroflexota* representatives (genera *Chloroflexus*, *Kallotenue*, *Caldilinea*). *Chloroflexus* species exhibit metabolic versatility, capable of photoheterotrophic and chemoheterotrophic growth [51,52,53]. In contrast, *Kallotenue papyrolyticum* is an aerobic heterotroph [54], while *Caldilinea* species grow chemoorganoheterotrophically under both aerobic and anaerobic conditions [55,56]. A subdominant part of the microbial communities of the T group was represented by heterotrophic bacteria of the phyla *Acidobacteriota*, *Bacteroidota*, *Pseudomonadota*, *Bacillota*, and others. Thus, the T group includes microbial communities that carry out mineralization of organic matter under aerobic and microaerobic conditions.

The MT group originated from both phototrophic mats and underlying sediments at different depths. Thus, the alpha diversity of the MT group microbial communities varied substantially, reflecting their specific locations within the stratified biogeocenosis. Phototrophic microbial mats characterized by low taxonomic diversity consisted of both aerobic phototrophs of the phylum *Cyanobacteriota* and bacteria of the phylum *Chloroflexota* carrying out anoxygenic photosynthesis or growing photoheterotrophically. Cyanobacteria likely function as primary producers of autochthonous organic matter in hot springs. Among phototrophic *Chloroflexota*, the most abundant groups belonged to the genera *Roseiflexus* and *Chloroflexus*. *Roseiflexus* can grow photoheterotrophically and chemoheterotrophically under anaerobic light and aerobic dark conditions, respectively, but neither photoautotrophic nor fermentative growth is observed [57]. Representatives of the genus *Chloroflexus* are capable of photoautotrophic growth, photoheterotrophic growth in the absence of oxygen, and chemoheterotrophic growth in aerobic conditions [51,52,53].

A green microbial mat from the Goryachinsk field was dominated by uncultured *Thiobacillaceae* (65.7%), likely oxidizing sulfur compounds chemolithoautotrophically, and *Chloroflexus* (20.2%). The underlying sediment microbial community exhibited high alpha diversity, and probably these microorganisms are responsible for the destruction of organic matter under anaerobic conditions. Significant microbial taxa of the MT group included *Dissulfurispiraceae*, uncultured *Nitrospirota* (SM23-35), and *Desulfobacterota* (BSN033), suggesting their role in the terminal degradation of organic matter and reducing the sulfate or other sulfur compounds to form sulfide.

M-group included four microbial communities from the Umkhei, Kuchiger and Zmeinaya Bay thermal springs with the lowest temperature ranging from 32 to 42 °C and significant anthropogenic effect. Both thermophilic taxa described above for the MT group and mesophilic bacteria were observed in these communities, resulting in quite a high taxonomic diversity. For example, the presence of the mesophilic sulfur-oxidizing bacteria of the genera *Thiocapsa*, *Lamprobacter*, *Thermochromatium*, *Thiobacillus*, *Thioflexithrix*, and *Thiothrix* has been shown for the Umkhey thermal springs. In addition, representatives of the genus *Aeromonas* and uncultivated representatives of the family *Rhodocyclaceae* were identified. For Umkhei and Kuchiger thermal springs, the presence of *Enterobacteriaceae* bacteria, primarily of *Enterobacter* and *Serratia* representatives, has been established, indicating a significant anthropogenic impact on these springs.

A significant share of archaea was detected only in sediments of the Kuchiger (#4705) and Gusikha (#4730) hot springs. *Archaea* were represented by uncultivated class *Bathyarchaeia* (*Thermoproteota*), the family *Hadarchaeaceae* (*Hadarchaeota*), as well as unclassified representatives of the phylum *Thermoplasmatota*. Most probably, archaea of the class *Bathyarchaeia* and the phylum *Thermoproteota* participate in organic matter decomposition [58,59,60,61]. For representatives of the phylum *Hadarchaeota* it was shown the ability to oxidize carbon monoxide coupled with nitrite reduction to ammonia [62], or methanogenic degradation of hydrocarbons mediated by syntrophic cooperation between archaeal partners [63].

### 4.2. Comparative Analysis of the Microbial Communities Studied with Those from Alkaline Hot Springs Worldwide

Thermal alkaline hot springs, which are widely distributed globally, are presented by two main groups: volcanic and non-volcanic. Volcanic hot springs are a characteristic of volcanically active regions, such as Yellowstone (USA), Iceland, Italy, or rift arcs associated with subduction along tectonic plate boundaries, such as the countries in the Pacific Ring of Fire (Japan, New Zealand, Chile, Costa Rica, etc.). These regions are characterized by a bimodal distribution of pH values ranging from acidic to alkaline. The bimodal pH distribution is a geological phenomenon that occurs due to subsurface boiling and vapor–liquid separation in geothermal systems when water is heated underground. The vapor phase is enriched with volcanic gases, such as hydrogen sulfide (H_2_S), which oxidizes in the presence of oxygen to form sulfuric acid (H_2_SO_4_), creating acidic, sulfate-rich springs. The liquid phase, which has lost volatile components, is neutral or alkaline and typically rich in sodium, chloride, and sulfate. The waters of this type of spring are heated by direct contact with magma chambers or bodies close to the surface [64,65,66,67,68]. Non-volcanic alkaline hot springs are found primarily in areas with deep crustal fractures, high radiogenic heat production, granitic rock masses, or along major fault lines, continental rift zones African (Kenya), Baikal (Russia), parts of the continental shield (India and China), or fault zones (for example, Spain, Romania, and Croatia). Their distribution is controlled by deep circulation of groundwater along major faults and permeable fracture networks that heat the water through contact with hot rocks. They are typically rich in potassium, sodium, and bicarbonates and have lower temperatures than volcanic springs because of a slower and more diffuse heating process [69,70,71,72,73,74].

An analysis of the literature on the diversity of microbial communities in alkaline springs has shown that, despite differences in the formation of volcanic and non-volcanic alkaline thermal springs, the main taxonomic composition of microbial communities within the four highlighted groups (ET, T, MT, and M-groups) is similar worldwide, regardless of the geology or cation and anion composition of the water. Similar microbial communities have been found in alkaline hot springs in the USA [65,75,76,77,78,79], Iceland [65], Italy [67], Japan [80], New Zealand [64], Indonesia [81]; Thailand and Philippines [82], Costa Rica [83]; Kenya [69], China [84,85,86,87,88,89], India [90,91,92,93,94,95,96,97,98,99], Romania [70], and Croatia [73]. In addition, a hyperthermophilic (HT) group of microbial communities can also be distinguished that are present in hot springs with temperatures above 80 °C and are characterized by a high share of bacteria of the phylum *Aquificota* and hyperthermophilic archaea of the phylum *Thermoproteota* [64,87,88,89,100,101]. It is also worth mentioning the unusual microbial communities, which are characterized by the dominance of various mesophilic taxa, regardless of the physicochemical factors in the alkaline hot springs [102,103,104]. We suggest that these unusual communities are an artifact.

### 4.3. Diversity of the Alkalithermophilic Dissimilatory Iron Reducers in BRZ

Analysis of primary enrichment cultures with ferrihydrite obtained previously from water and sediment samples of the Goryachinsk thermal field revealed an increase in the relative abundance of phylotypes belonging to phyla *Nitrospirota*, *Pseudomonadota*, and *Bacillota*, as well as less abundant phyla *Actinomycetota*, SVA0045, *Bacteroidota*, *Desulfobacterota*, *Deinococcota,* and *Thermotogota* [22]. Following several transfers under a molecular hydrogen atmosphere, the enrichment culture of alkalithermophilic iron reducers dominated by the phylotype closely related to *Parvivirga hydrogeniphila*, which comprised 52.3% of the community, was obtained [22]. *P. hydrogeniphila* is a thermophilic anaerobe and obligate autotrophic iron reducer that uses only H_2_ or formate as an electron donor, isolated from the aquifer of the Yessnetukskoye mineral water basin [105,106]. Interestingly, the type strain of this species cannot grow at pH values above 8.5 [105]. Based on these findings, the search for dissimilatory alkalithermophilic iron reducers was expanded in other hot springs of BRZ. To exclude fermentation processes, only non-fermentable substrates were used — molecular hydrogen or sodium acetate. The target microorganisms were detected in five samples out of seven tested, except for samples #4710 (Garga thermal field) and #4725 (Gusikha thermal field). The preferred donor for dissimilatory alkalithermophilic iron reducers was molecular hydrogen. Under selective conditions, the taxonomic composition of the communities shifted significantly from the microbial profiles observed in natural samples. 16S rRNA gene-based profiling revealed that the relative abundance of representatives from *Bacillota*, *Deinococcota*, and *Actinomycetota* phyla strongly increased in selective media designed to isolate alkalithermophilic dissimilatory iron reducers (Figure 5). This microbial profile was significantly different from the pattern of the natural samples, where *Bacillota* representatives constituted only a minor part of the communities (Figure 2). It is important to note that among these three phyla, the *Bacillota* was represented by the most diverse phylotypes. In contrast, the other two phyla were dominated almost exclusively by phylotypes belonging to only three genera: *Thermus*, *Meiothermus* (phylum *Deinococcota*), and *Parvivirga* (phylum *Actinomycetota*).

Representatives of the genus *Meiothermus,* as previously reported for the enrichment cultures from the Goryachinsk thermal field [22]. In addition, sequences related to the genus *Meiothermus* have been detected in the subsurface of hyperalkaline and suboxic environments, oftentimes in high relative abundance [107,108,109]. The representation of the reads related to this genus increased in all enrichment cultures compared to natural samples (Figure 6), except in sample #4730 and the enrichment culture from this spring (Gusikha thermal field). In both cases, phylotypes belonging to *Meiothermus* were not detected. It is also worth noting that in the third transfer of enrichment culture #4733, both with formate and acetate, this bacterium completely disappeared (Figure 6). Therefore, it can be assumed that representatives of this genus likely act as organotrophs in enrichments, decomposing organic matter obtained from natural samples rather than participating in the decomposition of mort mass. As for the phylotypes belonging to the genus *Thermus* in the enrichment culture SF4722.H2 (Uro thermal field), their functional role remains open to interpretation. Perhaps representatives of this genus may be involved in iron reduction, as this metabolic capability has been previously demonstrated for members of this genus [110,111].

The discovery and significant accumulation of a phylotype belonging to the *Parvivirga* genus (*Actinomycetota*) in enrichment cultures SF4730.H2 and SF4733.H2 support several important conclusions. First, this study confirms the previously detected presence of this bacterium in the water and sediments of the Goryachinsk thermal field [22], indicating that it is likely a permanent member of this microbial community. Second, its detection in the enrichment cultures SF4730.H2 from sediments of Gusikha thermal spring indicates a wider distribution of this genus in the BRZ. Third, its significant accumulation in a medium with ferrihydrite and molecular hydrogen confirms the previously identified narrow ecological specialization of this microorganism [105,106]. As described in our previous work, the phylotypes of this genus were not detected in any of the natural samples studied [22]. This also indirectly suggests that the surface conditions are not optimal for this microorganism, which is sensitive to redox potential and obligately dependent on hydrogen and ferric iron compounds. Finally, its accumulation in samples with alkaline pH values expands the known limits of its ecological niche. Thus, the findings of current research suggest that representatives of the genus *Parvivirga*, most likely a new species, are polyextremophilic dissimilatory iron reducers that transform ferrihydrite by oxidation of molecular hydrogen at pH ≥ 8.5 and T ≥ 50 °C.

The most diverse phylotypes whose abundance significantly increased in the presence of ferrihydrite with hydrogen or acetate belonged to the phylum *Bacillota*. In the native samples, their relative abundance ranged from 0.9 (sample #4722) to 5.48% (sample #4733), while in the enrichments it ranged from 18.7 (sample SF4732.H2) to 82.25% (sample SF4730.H2) (Figure 6). At the same time, a significant part of the detected phylotypes belonged to unknown bacteria of various taxon levels—from the genus to the class, for which no reliable data about physiology exist. In the enrichment cultures SF4722.H2 (Uro thermal field), SF4732.acetate, and SF4733.H2 (Goryachinsk thermal field), a significant accumulation of phylotypes belonging to the order *Desulfotomaculales* according to GTDB_r220_v2 was detected. However, BLAST analysis revealed a low similarity to all known genera within this order, suggesting they represent a taxon of a higher level. The V3-V4 hypervariable region of the 16S rRNA gene used in high-throughput sequencing provides insufficient phylogenetic resolution to classify this group further. The significant accumulation of these bacteria in the third transfer from native sample #4733 allowed us to suggest that their cells have a coccoidal morphology (Figure 7), which is unusual for representatives of the order *Desulfotomaculales*. Their high accumulation in the enrichment cultures from different thermal fields (Uro and Goryachinsk) indicates that they were not accidentally detected in the microbial communities of the BV hot springs despite being undetectable in the native samples by molecular methods. This phenomenon also suggests that these organisms thrive in the selective conditions, and, therefore, most likely, they are chemoorganotrophic or chemolithotrophic iron reducers that prefer thermal environments with a high pH value.

In addition to unidentified *Bacillota* phylotypes, representatives of known taxa were also enriched. The most common phylotype belonged to the genus *Anoxybacillus*, a significant accumulation of which was recorded in all enrichments except for the sample SF4722.H2 from the Uro thermal field, and turned out to be quite unexpected. Moreover, this phylotype accounted for up to 20% in the enrichment culture SF4733.acetate(III), which was obtained after two successive transfers of the primary enrichment SF4733.H2. The only described representative of this genus is a facultatively anaerobic alkalophilic fermenting bacterium [112,113]. However, it has been demonstrated that some representatives of this genus are able to reduce Fe(II)EDTA-NO and Fe(III)EDTA simultaneously [114]. Perhaps the presence of ferrihydrite in the medium somehow stimulated the growth of this bacterium, for example, by dumping of extra electrons during the fermentation of complex organic compounds. In addition to *Anoxybacillus*, other phylotypes belonging to the genera *Carboxydocella* and *Dethiobacter* were also observed, for which the ability to reduce iron has been previously demonstrated [83,84,85,86]. According to BLAST analyses phylotype related to the genus *Carboxydocella* had 99.07% identity with *Carboxydocella manganica*, a thermophilic, anaerobic, dissimilatory Fe(III) and Mn(IV)-reducing bacterium isolated from a terrestrial hot spring on the Kamchatka peninsula. This is a neutrophilic bacterium with a pH range from 5.5 to 8.0, with an optimum at pH 6.5 [115]. Phylotype related to alkaliphilic iron- or thiosulfate-reducing bacteria of the mesophilic genus *Dethiobacter* [116] demonstrated a low (93%) level of similarity according to BLAST analysis. It should be noted that *Dethiobacter*-related phylotypes have been previously detected exclusively in alkaline environments (pH ≥ 7.5) of two different types: the sediments of soda or meromictic lakes, and subsurface ecosystems affected by serpentinization processes [117].

## 5. Conclusions

For the first time, a comprehensive analysis of polyextremophilic microbial communities in thermal springs in the Baikal Rift Zone was conducted using co-occurrence network and NMDS approaches, which allowed a reveal of patterns that determine their taxonomic composition. The alkaline pH values ranging from 7.8 to 9.5 showed low correlation with the structure of the studied microbial communities. In contrast, temperature turned out to be a critical factor determining the uniqueness of microbiomes in the thermal springs of the BRZ, directly impacting the alpha and beta diversity. Trophic structure of the studied communities included primary producers—aerobic and anaerobic phototrophs, sulfur-oxidizing autotrophs; aerobic and anaerobic organoheterotrophs—destructors of complex (polysaccharides, protein compounds, amino acids) and simple (carbohydrates, organic acids) organic compounds. The final stage of organic matter decomposition is most likely carried out by sulfate-reducing bacteria. The discovery of numerous uncultivated bacteria from deep phylogenetic lineages raises significant questions about their functional roles. In particular, these organisms can use alternative terminal acceptors besides sulfate for organic matter mineralization. Metagenomic analysis, as well as cultivation on selective media, will help clarify this issue. The latter method was successfully applied here to identify microorganisms that could transform iron minerals.

Our findings confirmed that iron-reducing microorganisms can transform iron minerals at high pH and temperature, as hypothesized by Nixon et al. [19]. Compared to our first work [22], we significantly expanded our knowledge of the phylogenetic diversity of this physiological group. Alkalithermophilic iron reducers are rare or not even detectable by molecular methods in the BRZ thermal freshwater microbial communities; however, our results revealed their ability to outcompete phototrophs and organotrophs rapidly and displace them effectively in selective media. As hydrogen has turned out to be a preferable substrate for this group, they may be more prevalent among subsurface microbial communities. This ecosystem is even more attractive to the alkalithermophilic iron reducers because it is depleted in organic matter but not limited in Fe(III) from Proterozoic igneous rocks and the overlying Quaternary sediments. The dominance of *Bacillota* representatives in the enrichment cultures suggests that chemolitho- and chemoorganotrophic members of this phylum are most likely responsible for the reduction of Fe(III) in alkaline freshwater thermal springs of BRZ.

## Figures and Tables

**Table 1:** General characteristics of the BRZ hot springs and alpha diversity metrics of microbial communities there.

Sample	T, °C	pH	Eh, mV	Coordinates	Number of ASVs Detected	Number of ASVs with More than 1% Abundance	Chao1	Shannon	InvSimpson
4703	32	7.6	−238	54.987511 111.118274	427	16	427	4.845	45.739
4704	42	9.5	−330	54.987872 111.118483	334	21	334	4.365	33.301
4705	41	6.9	NA	54.882258 111.001	281	25	281	4.401	39.919
4707	74	7.8	−190	54.32022 110.993979	42	18	43	2.641	8.745
4708	74	7.8	−190	54.32022 110.993979	23	6	23	1.608	3.241
4709	74	7.8	−190	54.32022 110.993979	68	15	68	2.334	4.780
4711	72	7.9	−160	54.320076 110.993912	76	16	76	2.583	5.006
4715	59	8.2	5	54.319995 110.993898	204	25	204	3.757	22.915
4718	42	9.0	20	53.44499 110.11831	118	4	118	1.929	4.601
4719	48	9.0	20	53.444944 110.118236	139	15	139	2.857	8.205
4721	50	9.0	−80	53.444934 110.118312	253	12	253	4.123	26.826
4722	59	9.0	−40	53.444934 110.118312	79	15	79	2.966	11.746
4723	59	8.8	−50	53.444934 110.118312	85	10	85	1.873	2.626
4724	58	9.0	10	53.445062 110.118303	39	11	39	1.710	2.975
4725	50	9.0	−200	53.444934 110.118312	277	18	277	4.286	37.994
4728	53	8.3	55	53.414930 109.354914	211	24	211	3.790	15.816
4730	51	9.1	−115	53.414989 109.354720	227	17	227	3.734	17.388
4731	52	9.1	−115	52.987259 108.30778	125	17	125	3.166	12.368
4732	45	7.2	10	52.987238 108.307715	231	12	231	3.593	15.933
4733	48	9.0	−170	52.987238 108.307715	389	18	389	4.480	33.697
4734	48	9.0	−300	52.987188 108.307606	72	6	72	1.575	2.331
4735	48	7.9	−160	52.987188 108.307606	515	22	515	5.194	76.778
4736	41	9.3	−350	53.766396 109.027353	176	12	176	2.838	6.318

## Data Availability

All sequencing data were deposited into the NCBI SRA database under BioProject number PRJNA1335199.

## Supplementary Materials

The following supporting information can be downloaded at: https://www.mdpi.com/article/10.3390/biology14121716/s1, Supplementary Material S1, Supplementary Material S2: Description of the sampling sites.

## Abbreviations

The following abbreviations are used in this manuscript:

ASVs	Amplicon sequence variations
BLAST	Basic Local Alignment Search Tool
BRZ	The Baikal Rift Zone
BV	The Barguzin Valley
ET group	The Extreme Thermophilic group
GTDB	The Genome Taxonomy Database
M group	The Mixed group
MT	The Moderate Thermophilic group
ND	Not detected
NMDS	Non-metric multidimensional scaling
T group	Thermophilic group
