# Supplementary material for: Microbial Diversity of the Baikal Rift Zone Freshwater Alkaline Hot Springs and the Ecology of Polyextremophilic Dissimilatory Iron-Reducing Bacteria
Source: Biology (Basel). 2025 Dec 1;14(12):1716. doi: 10.3390/biology14121716 (PMC12730654; doi:10.3390/biology14121716)
Supplement: Supplementary file 1 [file biology-14-01716-s001.zip › Supplementary Materials 2_corrected.pdf]

**Supplementary Materials to**

# Microbial diversity of the Baikal Rift Zone freshwater alkaline hot springs and the ecology of polyextremophilic dissimilatory iron-reducing microorganisms

**Supplementary Table 1.** Main characteristics of the sampling sites. DL, detection limit; ND – no date.

| Characteristics | Umkhei | Kuchiger | Garga | Uro | Gusikha | Goryachinsk | Zmeiny |
|---|---|---|---|---|---|---|---|
| T, °C | 41 – 48 | 38 – 57 | 74 – 77 | 29 – 71 | 43 – 73 | 46 – 56 | 37 – 46 |
| pH | 9.2 – 10.1 | 9.0 – 10.2 | 7.6 – 8.3 | 8.4 – 9.7 | 8.2 – 8.6 | 8.8 – 9.7 | 9.4 – 9.6 |
| Eh, mV | –410 – –50 | –408 – –300 | +35 – +113 | +80 – +86 | –72 – +96 | –60 – –206 | –438 – –350 |
| Water-bearing rocks: | | | | | | | |
| Rocks | Granites and limestones | Granites | Granites | Biotite granites and granosyenite | Granites, granodiorites and granosyenites | Gneisses and amphibolites intruded by intrusive complexes | Granite gneisses |
| Age | $\gamma PR_2$ | $\gamma PR_2$ | $\gamma PR_2$ | $\gamma\xi C_{2-3}$ | $\gamma PR_2$ | $PR_1$-$AR_2$ | $\gamma PR_2$ |
| Chemical composition of the thermal waters, mg $L^{-1}$: | | | | | | | |
| Type | Sodium hydrocarbonate–sulfate | Sodium hydrocarbonate–sulfate | Sodium sulfate | Sodium sulfate or sodium hydrocarbonate–sulfate | Sodium sulfate | Sodium sulfate | Sodium sulfate |
| TDS | 156.0 – 506.0 | 346.0 – 465.0 | 700.0 – 1065.0 | up to 345.0 | 640.5 – 858.0 | 491.0 – 685.0 | 359.2 – 582.0 |
| $Na^+$ | 96.0 – 148.6 | 101.0 – 118.0 | 263.0 – 315.0 | 77.0 – 96.0 | 169.0 – 211.0 | 129.0 – 155.0 | 108.0 – 146.0 |
| $K^+$ | 0.80 – 1.76 | 0.90 – 1.51 | 9.0 – 18.3 | 2.9 – 25.0 | 7.0 – 12.0 | 2.0 – 4.3 | 1.29 – 3.22 |
| $Ca^{2+}$ | 0.9 – 5.2 | 1.1 – 8.0 | 23.0 – 27.1 | 6.5 – 6.6 | 13.5 – 17.2 | 8.1 – 34.7 | 2.9 – 4.7 |
| $Mg^{2+}$ | 0.03 – 1.00 | 0.03 – 19.46 | 0.04 – 1.10 | 0.01 – 0.43 | 0.10 – 0.98 | 0.03 – 2.44 | 0.01 – 0.24 |
| $HCO_3^-$ | 24.0 – 134.0 | 22.0 – 110.0 | 92.0 – 112.0 | 50.0 – 85.4 | 80.0 – 106.0 | 7.3 – 42.0 | 68.4 – 144.0 |
| $CO_3^{2-}$ | 3.0 – 43.0 | 19.5 – 60.0 | 0 – 6.0 | 0 – 18.0 | 0 – 9.4 | 6.6 – 15.6 | 25.2 – 42.0 |
| $SO_4^{2-}$ | 55.8 – 116.0 | 70.9 – 201.0 | 390.0 – 490.9 | 35.8 – 150.0 | 272.9 – 356 | 245.7 – 365.0 | 122.0 – 151.0 |
| $Cl^-$ | 10.3 – 23.5 | 7.1 – 34.1 | 37.5 – 56.1 | 10.6 – 14.0 | 31.0 – 36.4 | 6.2 – 14.8 | 23.0 – 30.5 |
| $SiO_2$ | 68.4 – 95.0 | 80.8 – 97.0 | 58.7 – 91.0 | 96.0 – 118.0 | 88.3 – 103.0 | 60.9 – 79.0 | 88.0 – 111.0 |
| $F^-$ | 14.4 – 15.8 | 10.9 – 13.9 | 10.5 – 12.0 | 5.9 – 7.5 | 9.5 – 10.9 | 3.0 – 3.4 | 9.1 – 10.6 |
| $S^{2-}$ | up to 31.0 | up to 47.9 | 0 | 0 | up to 4.4 | trace | up to 1.8 |
| Microelement composition of water, μg $L^{-1}$: | | | | | | | |
| Al | 59.87 | 120.72 | 33.7 | 42.16 | 37 | 21 | 38-30 |
| As | 0.0388 | 0.0648 | 0.0877 | 4.454 | 0.14 | 0.05 | 0.15-0.13 |
| B | 134.32 | 159.70 | 260.82 | 166.0 | ND | ND | ND |
| Ba | 1.757 | 4.544 | 46.25 | 3.845 | 35.86 | 5.65 | 1.28-3.27 |
| Be | <DL | <DL | 0.6023 | 0.584 | 0.078 | 0.036 | 0.029-0.045 |
| Cr | 0.1237 | 1.054 | 0.2082 | 2.505 | 4.74 | 2.84 | 0.93-1.02 |
| Cs | 1.060 | 1.636 | 42.85 | 8.780 | 6.73 | 5.42 | 1.87-3.10 |
| Cu | <DL | <DL | 1.306 | <DL | 31.6 | 51.1 | 10.1-32.2 |
| Fe | 35.28 | 153.6 | 16.67 | 23.211 | ND | ND | ND |
| Ga | 5.934 | 6.261 | 3.861 | 4.315 | 1.52 | 1.20 | 4.15-4.11 |
| Ge | 8.018 | 10.12 | 10.61 | 2.668 | 1.97 | 1.05 | 6.44-4.96 |
| Li | 124.72 | 91.46 | 1736 | 100.91 | 163 | 42 | 26-45 |
| Mn | 2.278 | 6.184 | 13.30 | 1.294 | 9.5 | 4.6 | 6.8-4.8 |
| Mo | 0.7188 | 1.380 | 14.35 | 49.58 | 16.42 | 4.68 | 0.51-0.77 |
| Ni | 0.2288 | 0.3737 | 0.4905 | 0.1830 | 11.34 | 7.02 | 2.3-8.97 |
| P | 98.20 | 15.13 | 92.40 | 64.25 | ND | ND | ND |
| Pb | 0.3014 | 0.3781 | 0.4948 | 0.2540 | 11.9 | 1.8 | 2.7-3.7 |
| Rb | 14.53 | 12.03 | 118.59 | 28.26 | 56.4 | 14.6 | 13.6-22.6 |
| Se | <DL | 0.0614 | <DL | <DL | ND | ND | ND |
| Sn | 0.0361 | 0.2320 | 0.1240 | 0.045 | 0.55 | 0.05 | 0.02-0.16 |
| Sr | 136.28 | 397.41 | 3794 | 237.38 | 1051 | 462 | 90-249 |
| Ti | 1.585 | 3.613 | 0.8597 | 1.437 | 4.7 | 4.5 | 3.0-4.3 |
| U | 0.0109 | 0.0747 | 0.0056 | 0.3179 | 8.63 | 0.06 | 0.03-0.04 |
| V | 0.2391 | 0.7396 | 0.0405 | 2.505 | 1.4 | 0.43 | 0.47-0.44 |
| W | 8.641 | 9.3981 | 80.82 | 75.23 | 30.0 | 16.7 | 11.8-29.7 |

| | | | | | | | |
|---|---|---|---|---|---|---|---|
| Zn | 3.374 | 15.83 | 6.144 | 3.376 | 121 | 47.8 | 9.1-28.2 |
| Zr | 0.0323 | 0.0640 | 0.0525 | <DL | 1.06 | 0.84 | 0.15-0.43 |
| Gas concentrations, vol. %: | | | | | | | |
| $N_2$ | 92.2 - 98.7 | 27.6 – 37.9 | 83.4 | 98.0 | 89.5 – 90.2 | 91.9 | 91.4 – 98.9 |
| $CH_4$ | 0.17 - 0.22 | 60.9 – 62.2 | 0.8 | ND | 0.01 – 0.02 | 1.1 | 0.14 – 0.29 |
| $CO_2$ | <0.01 | <0.01 | ND | ND | up to 2.92 | 1.0 | <0.01 |
| $H_2$ | <0.004 | <0.004 | ND | ND | <0.004 | 0 | up to 0.016 |
| $O_2$ | 0.51 - 0.93 | 0.58 – 1.59 | up to 8.4 | ND | up to 7.1 | 4.0 | 0.45 – 0.58 |
| He | 0.12 | 0.02 – 0.03 | ND | ND | 0.08 – 0.11 | 0.1 | 0.15 – 0.16 |
| References | [1,5,7,16,17,30-32] | [1,5,15-17,31,32] | [1,2,5,9,12] | [1,5,11] | [1,5,32,] | [1,5,14,32,] | [1,32,87] |

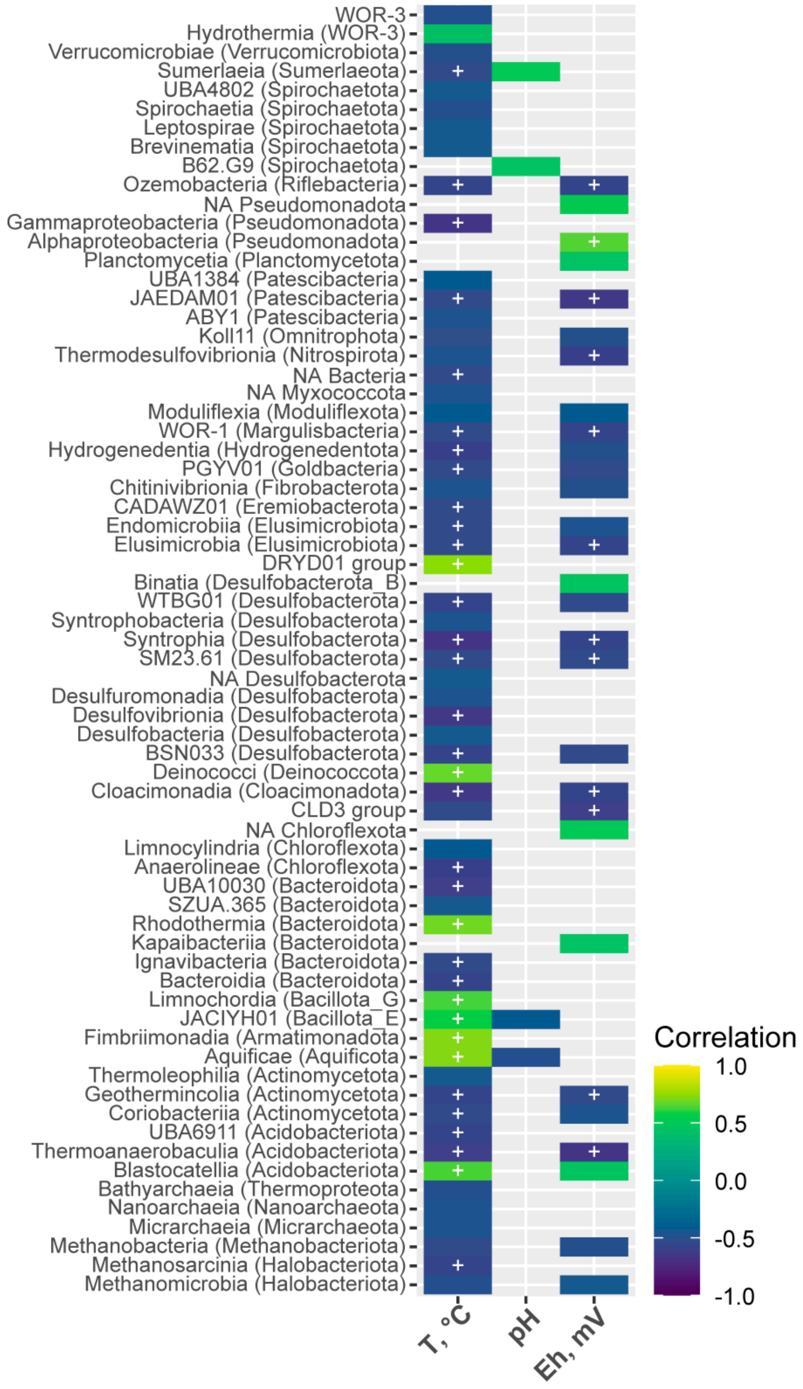


**Supplementary Figure 1.** Spearman's rank correlations between the relative abundances of the prokaryotic classes and the environmental variables (temperature, pH and redox potential). Only significant pairwise correlations are shown (p-value < 0.05); "+" indicate p-values < 0.01.
